# Investigation of the Activity of the Microorganisms in a Reblochon-Style Cheese by Metatranscriptomic Analysis

**DOI:** 10.3389/fmicb.2016.00536

**Published:** 2016-04-20

**Authors:** Christophe Monnet, Eric Dugat-Bony, Dominique Swennen, Jean-Marie Beckerich, Françoise Irlinger, Sébastien Fraud, Pascal Bonnarme

**Affiliations:** ^1^UMR Génie et Microbiologie des Procédés Alimentaires, AgroParisTech, INRA, Université Paris-SaclayThiverval-Grignon, France; ^2^Actalia Produits LaitiersLa Roche-sur-Foron, France

**Keywords:** cheese, metatranscriptome, RNA-seq, reverse transcription-quantitative PCR, gene expression profiling, *Geotrichum candidum*, *Debaryomyces hansenii*

## Abstract

The microbial communities in cheeses are composed of varying bacteria, yeasts, and molds, which contribute to the development of their typical sensory properties. *In situ* studies are needed to better understand their growth and activity during cheese ripening. Our objective was to investigate the activity of the microorganisms used for manufacturing a surface-ripened cheese by means of metatranscriptomic analysis. The cheeses were produced using two lactic acid bacteria (*Streptococcus thermophilus* and *Lactobacillus delbrueckii* ssp. *bulgaricus*), one ripening bacterium (*Brevibacterium aurantiacum*), and two yeasts (*Debaryomyces hansenii* and *Geotrichum candidum*). RNA was extracted from the cheese rinds and, after depletion of most ribosomal RNA, sequencing was performed using a short-read sequencing technology that generated ~75 million reads per sample. Except for *B. aurantiacum*, which failed to grow in the cheeses, a large number of CDS reads were generated for the inoculated species, making it possible to investigate their individual transcriptome over time. From day 5 to 35, *G. candidum* accounted for the largest proportion of CDS reads, suggesting that this species was the most active. Only minor changes occurred in the transcriptomes of the lactic acid bacteria. For the two yeasts, we compared the expression of genes involved in the catabolism of lactose, galactose, lactate, amino acids, and free fatty acids. During ripening, genes involved in ammonia assimilation and galactose catabolism were down-regulated in the two species. Genes involved in amino acid catabolism were up-regulated in *G. candidum* from day 14 to day 35, whereas in *D. hansenii*, they were up-regulated mainly at day 35, suggesting that this species catabolized the cheese amino acids later. In addition, after 35 days of ripening, there was a down-regulation of genes involved in the electron transport chain, suggesting a lower cellular activity. The present study has exemplified how metatranscriptomic analyses provide insight into the activity of cheese microbial communities for which reference genome sequences are available. In the future, such studies will be facilitated by the progress in DNA sequencing technologies and by the greater availability of the genome sequences of cheese microorganisms.

## Introduction

Many types of fermented foods are produced throughout the world. Fermentation improves the shelf life of these products and contributes to their typical sensory and nutritional properties. Cheeses are fermented dairy products whose manufacturing involves different types of bacteria, yeasts, and molds (Montel et al., 2014; Irlinger et al., 2015). Cheese manufacturing can be considered as a process in which a nutrient-rich environment, milk, is colonized by adventitious and deliberately inoculated microorganisms. Two different habitats may be considered, the interior of the cheese and the cheese rind. The rind microbiota can be considered as an interesting model system for the field of ecosystems biology (Wolfe et al., 2014). A better understanding of the activity of cheese rind microbiota would also be useful for cheesemakers, who are frequently faced with problems of unstable ripening activity or of growth of undesirable microorganisms in surface-ripened cheeses.

For a long time, our knowledge concerning the activity of cheese microbiota resulted, to a large extent, from studies involving biochemical analyses of cheeses during ripening, in which it was difficult to disentangle the contribution of the different microbial components. However, the development of metagenomic, metatranscriptomic, and metaproteomic approaches offers considerable perspectives (Ndoye et al., 2011; Cocolin and Ercolini, 2015). Recent technical advances and cost reduction of high-throughput sequencing technologies have made RNA sequencing (RNA-seq) available to researchers for metatranscriptomic studies, and several studies concerning food microbiota were recently published (Jung et al., 2013; Bisanz et al., 2014; Lessard et al., 2014; Dugat-Bony et al., 2015; Wang et al., 2015). Varying questions concerning the microbial physiology during cheese manufacturing may be addressed using metatranscriptomic analyses. One of these is the assessment of the energy substrates that are used by the dominant microbial species and the changes in energy substrates that occur during ripening. In addition, metatranscriptomic analyses may help to better understand how cells cope with the changes in pH, temperature, and salt and oxygen content, which may occur during cheese manufacturing. Lessard et al. (2014) monitored the activity of the yeast *Geotrichum candidum* and the mold *Penicillium camemberti* in the rind of Canadian Camembert-type cheeses. Long sequencing reads were generated, assembled *de novo*, and the resulting contigs were submitted to functional assignment. In another study, the RNA from the rind of a model surface-ripened cheese was shotgun sequenced by short-read sequencing, a technique that can generate tens of millions of sequencing reads (Dugat-Bony et al., 2015). The sequences were then mapped on the reference genomes of the inoculated strains to generate the metatranscriptomes. These two studies demonstrated the feasibility and benefits of RNA metatranscriptomic analysis of cheese microbiota. However, in order to obtain more information from cheese metatranscriptome analyses, it would be important to increase the number of mRNA sequencing reads. Indeed, this would make it possible to generate individual transcriptomes of the species in which a large proportion of genes are detected and covered with sufficient reads to perform statistical comparisons of expression among different samples. The objective of the present study was to investigate a cheese rind microbiota using a metatranscriptomic approach whose efficiency is sufficient to identify transcriptional regulations in order to better characterize important metabolic features during cheese ripening such as changes in energy substrates. For that purpose, we depleted fungal and bacterial rRNA from the RNA samples, and performed short-read sequencing in order to generate at least 50 million reads per sample. We investigated Reblochon-style cheeses that were previously manufactured for a study in which the activity of several *G. candidum* genes was measured by real-time PCR (Castellote et al., 2015). Reblochon is a French cheese whose ripening is mainly determined by the activity of the rind. It contains lactic acid bacteria such as *Streptococcus thermophilus* and *Lactobacillus delbrueckii* ssp. *bulgaricus*, yeasts such as *G. candidum* and *Debaryomyces hansenii*, and aerobic ripening bacteria such as *Brevibacterium* (Cogan et al., 2014).

## Materials and methods

### Cheesemaking

The production and sampling of the cheeses investigated in the present work were previously described in Castellote et al. (2015). The typical Reblochon manufacturing process was used, except that pasteurized milk was used instead of raw milk. The rinds of three separate cheeses (referred to as “cheese replicates”) were sampled at day 5, 14, 19, and 35. The upper and lower parts of the cheese samples were removed with a knife (thickness: ~2–3 mm), pooled, rapidly homogenized with a garlic press, and immediately used for RNA extraction. DNA extraction was performed later using the cheese rinds that were stored at –30°C.

### Extraction of DNA from cheese samples and 16S gene rRNA metabarcoding analysis

Cheese samples were diluted 1:10 (w/v) in sterile distilled water and homogenized with an Ultra Turrax® (Labortechnik) at 8000 rpm for 1 min. DNA extraction was performed on 0.5 g of the mixture using the bead beating-based protocol detailed in a previous study (Dugat-Bony et al., 2015). DNA concentration was determined using a Qubit 3.0 fluorimeter (ThermoFischer Scientific, Villebon-sur-Yvette, France) and the associated kit. PCR amplification, library preparation, and sequencing were performed at the GeT PlaGe platform (Toulouse, France). Briefly, the V3V4 region of the 16S rRNA gene was amplified from 10 ng of purified genomic DNA with the primers F343 (CTTTCCCTACACGACGCTCTTCCGATCTTACGGRAGGCAGCAG) and R784 (GGAGTTCAGACGTGTGCTCTTCCGATCTTACCAGGGTATCTAATCCT) using 30 amplification cycles with an annealing temperature of 65°C. Single multiplexing was performed using homemade 6 bp indexes, which were added during a second PCR with 12 cycles. The resulting PCR products were purified and loaded onto the Illumina (San Diego, CA, USA) MiSeq cartridge, according to the manufacturer's instructions. The quality of the run was internally checked using PhiX, and each paired-end sequence was then assigned to its sample with the help of the previously integrated index. Paired-end reads were assembled using Flash (Magoč and Salzberg, 2011) prior to further analyses. Fastq files were then quality-filtered and analyzed using QIIME v.1.9.0 (Caporaso et al., 2010). Chimeric sequences were identified by means of USEARCH (Edgar, 2010) implemented in QIIME's pipeline using both reference modes against the SILVA LTP database (release 119; Yarza et al., 2008) and the *de novo* mode. Operational taxonomic units (OTUs) were clustered at 97% identity using QIIME's *de novo* OTU-picking pipeline and UCLUST (Edgar, 2010). Representative sequences for each OTU were taxonomically classified using BLAST (Altschul et al., 1990) against the SILVA LTP database (release 119), and assignations were manually controlled using EzTaxon (Kim et al., 2012).

### Extraction of RNA from cheese samples and rRNA depletion

RNA was extracted from 500-mg rind sample aliquots without prior separation of microbial cells, as previously described (Monnet et al., 2012), except that the DNase treatment was performed on the RNeasy spin columns (Qiagen, Courtaboeuf, France). Purified RNA was quantified with Qubit RNA assay kits on the Qubit 3.0 fluorimeter (ThermoFischer Scientific). RNA quality was analyzed with an Agilent model 2100 Bioanalyzer (Palo Alto, CA, USA) using RNA 6000 NANO chips, according to the manufacturer's instructions. For each sample, 10 μg of total RNA was then subjected to rRNA depletion using the Epicentre Ribo-Zero™ Magnetic Gold Kit (Tebu-bio, Le Perray-en-Yvelines, France) for bacteria (reference MRZB12424) and for yeasts (reference MRZY1324). Depletion was performed according to the manufacturer's instructions, except that a mixture (50/50) of the yeast and bacteria Ribo-Zero rRNA solutions was used. The rRNA-depleted samples were then purified using a RNeasy MinElute Cleanup Kit (Qiagen), according to the modified procedure described in the Ribo-Zero™ Magnetic Gold Kit technical procedure. The quality of depleted RNA was assessed on an Agilent model 2100 Bioanalyzer, using RNA 6000 PICO chips.

### cDNA library construction, RNA sequencing, and mapping against reference genomes

RNA (50 ng) was fragmented using RNA fragmentation reagents (Ambion reference AM8740, ThermoFischer Scientific), according to the manufacturer's recommendations. They were then treated with antarctic phosphatase (NEB, Evry, France) and Polynucleotide Kinase (NEB). Strand-oriented libraries were prepared with the TruSeq™ Small RNA sample preparation kit (Illumina), the final gel purification step being replaced by a PCR cleanup with AMPure XP beads (Beckman Coulter, Villepinte, France). Library quality was assessed on an Agilent 2100 Bioanalyzer, using an Agilent High Sensitivity DNA Kit. Libraries were sequenced on an Illumina HiSeq1000 instrument, with a TruSeq SR Cluster Kit v3-cBot-HS (Illumina) and a TruSeq SBS v3-HS (50-cycle) kit (Illumina), using a Single Read 50 bp recipe. Libraries were pooled in equimolar proportions and diluted to a final concentration of 12 pM, according to Illumina recommendations. Sequencing reads were split according to barcode sequence with the Illumina Casava 1.8.2 pipeline to generate a fastq file for each sample. The sequencing reads were trimmed for the presence of Illumina adapter sequences using Cutadapt 1.3 (Martin, 2011), and reads with a size of <10 after trimming were discarded. The short read sequences were mapped against the reference database using TopHat2 v2.0.11 (Kim et al., 2013). A maximum of two mismatches was allowed for each sequencing read. The reference database was composed of the CDSs, tRNA, and rRNA sequences of strains belonging to the same species as the five microorganisms that were inoculated into the cheese: *S. thermophilus* LMD-9, *L. delbrueckii* ssp. *bulgaricus* ATCC 11842, *Brevibacterium aurantiacum* ATCC9174, *D. hansenii* CBS767, and *G. candidum* ATCC 204307 (NCBI BioProject accession numbers are PRJNA13773, PRJDB769, PRJNA405, PRJNA13832, and PRJEB5752 for LMD-9, ATCC 11842, ATCC 9174, CBS 767, and ATCC 204307, respectively).

### RNA-seq data analyses

Sequence reads mapping to the coding DNA sequence (CDS) features were retrieved from the raw dataset. Data were filtered to remove genes displaying an average of <10 reads per sample across the entire dataset (12 samples). Two types of normalization were performed using custom scripts built under the statistical environment R (http://www.r-project.org/): according to the library size (for each sample, the read numbers were divided by the sum of the reads that mapped to the CDSs of the complete database), and according to each species (for each sample, the read numbers were divided by the sum of the reads that mapped to the CDSs of the species). Functional classification of the metatranscriptomic dataset was performed using the Kyoto Encyclopedia of Genes and Genomes (KEGG) annotations (Kanehisa et al., 2012). Principal component analysis was performed using PAST software (Hammer et al., 2001). Differential expression analysis was conducted using the Bioconductor DESeq2 package in the statistical environment R (Gentleman et al., 2004; Love et al., 2014). Raw *p*-values were adjusted for multiple testing using the Benjamini-Hochberg procedure (Benjamini and Hochberg, 1995), which assesses the false discovery rate. Gene transcripts with an adjusted *p* < 0.05 were considered to be differentially expressed between two ripening times.

### Sequence accession numbers

The raw Illumina data for all the samples was deposited in the European Nucleotide Archive of the European Bioinformatics Institute under the accession number PRJEB11361.

## Results

### Sequencing of the RNA samples

**Figure 1:**
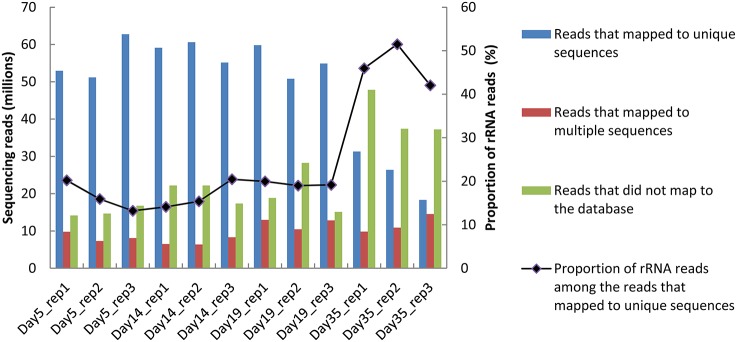
**Distribution of the sequencing reads generated from the 12 samples as a function of their mapping to the reference database**.

**Figure 2:**
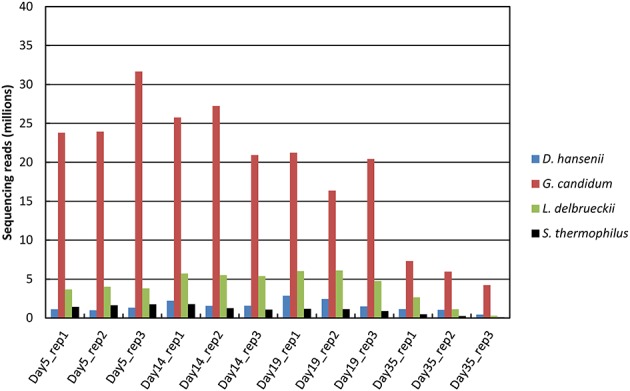
**Distribution of the sequencing reads that mapped to CDSs as a function of the reference genome**. Only reads that mapped to unique sequences of the database were considered. *Brevibacterium aurantiacum* reads were not represented because their number was very low (from 2000 to 27,000 reads, depending on the sample).

Between 70 and 91 million sequencing reads were generated from each of the 12 cheese rind samples, which corresponded to three cheese replicates that were analyzed at four sampling times. From day 5 to 19, most reads mapped to unique sequences of the reference database (Figure 1). At day 35, there was a higher number of reads that did not map to the database, which may be due to the lower RNA integrity at this sampling point (Castellote et al., 2015) and/or to the growth of other microorganisms. For the samples from day 5 to 19, the rRNA removal procedure was efficient, since only about 20% of the reads that mapped to unique sequences corresponded to ribosomal RNA. However, its efficiency decreased at day 35, when the rRNA reads accounted for 42 to 51% of the reads that mapped to unique sequences. In all of the samples, the number of reads that mapped to CDSs was the highest for *G. candidum* (from 4 to 32 million reads; Figure 2) and was very low for *B. aurantiacum* (from 2000 to 27,000 reads). The proportion of CDSs from the reference database that were detected (detection cut-off was set to a mean number of 10 reads per CDS and per sample) was 76, 87, 2, 88, and 96% for *S. thermophilus, L. delbrueckii, B. aurantiacum, D. hansenii*, and *G. candidum*, respectively. Sufficient reads were thus generated to study the transcriptome of all the inoculated strains, except for *B. autantiacum*. 16S rRNA gene metabarcoding analyses were performed on DNA samples from the cheese rinds in order to determine whether the low abundance of *B. aurantiacum* CDS reads was due to a poor growth of this species. The results showed that the relative proportion of *B. aurantiacum* among the bacterial community was very low (Supplementary Table 1). Microorganisms related to *Lactobacillus casei* that were not deliberately inoculated into the cheeses accounted for more sequencing reads than *B. aurantiacum*. The latter species grew thus quite poorly in the cheeses, which explains why very few of its CDSs were detected. The enumerations on agar plates performed by Castellote et al. (2015) on the cheese samples showed that, at day 35, the concentration of *S. thermophilus, L. delbrueckii* ssp. *bulgaricus, G. candidum*, and *D. hansenii* was 9.3, 7.3, 8.1, and 8.5 log_10_ cfu/g, respectively.

### Data normalization, analysis of cheese replicates, and correlation with quantitative PCR analyses

**Figure 3:**
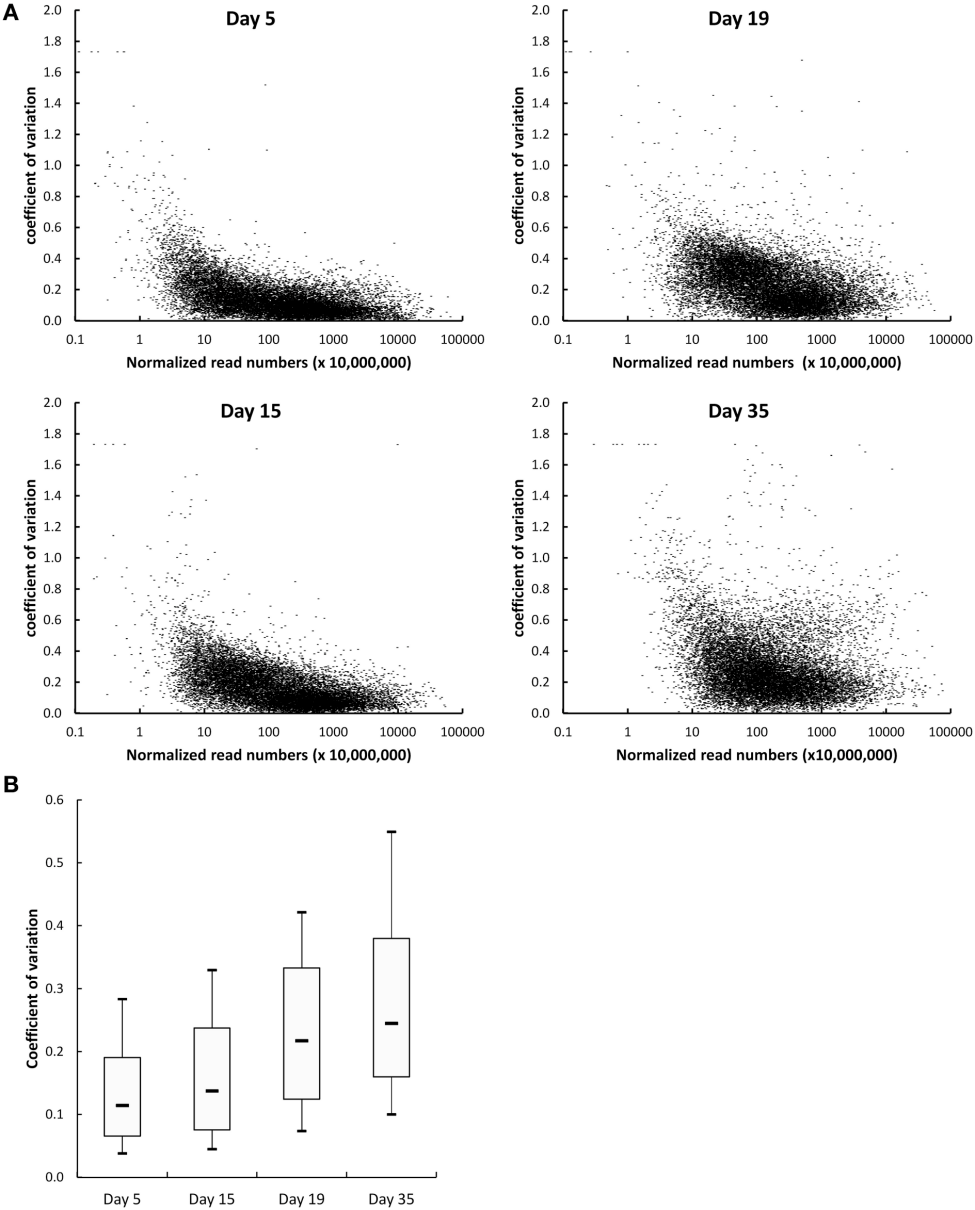
**Dispersion of the RNA-seq data generated from cheese replicates. (A)** Each dot represents one CDS. The coefficients of variation corresponding to the three cheese replicates were calculated using the data normalized against the complete database. **(B)** Distribution of the coefficients of variation of the normalized read numbers at the four sampling days. The boxplots represent the median values, the first and the third quartile, and the first and the 9th decile.

**Figure 4:**
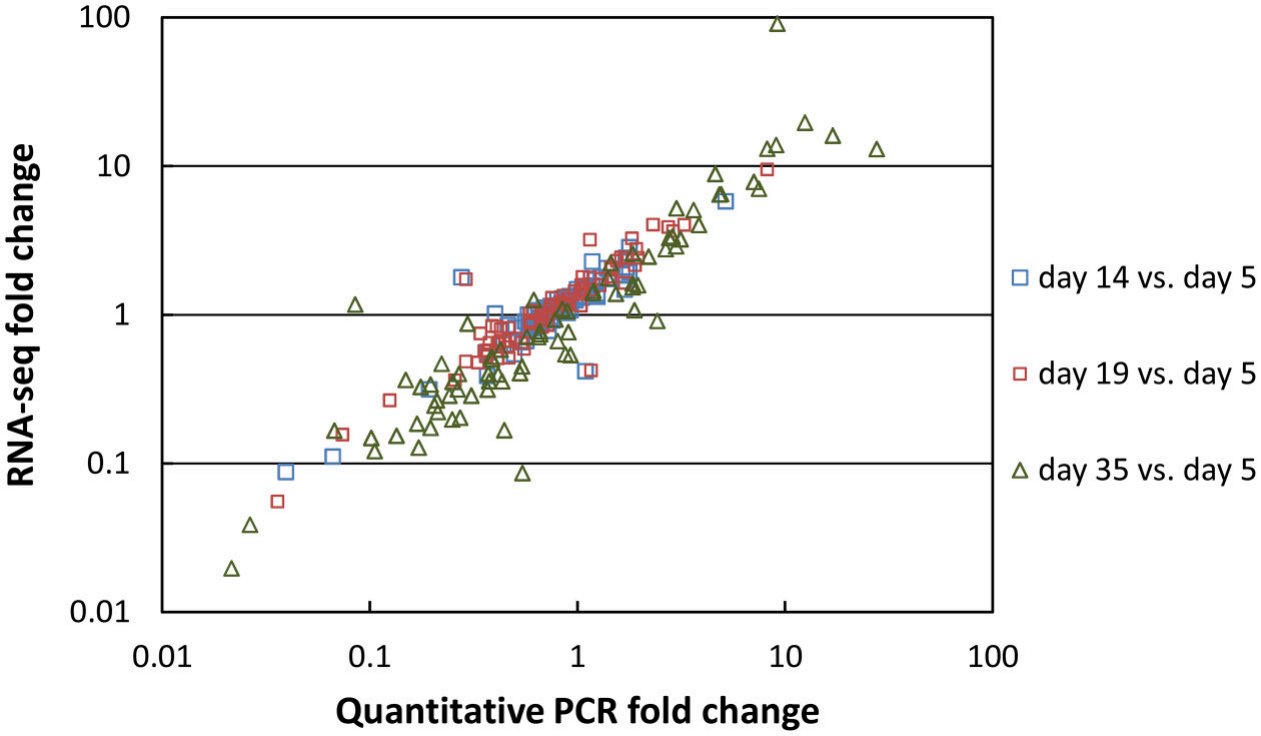
**Correlation between RNA-seq and reverse transcription-quantitative PCR data**. The fold changes vs. day 5 were calculated for 80 *G. candidum* genes.

Two types of normalization of the RNA-seq data were performed: against the complete database (read numbers were divided by the sum of the reads that mapped to the CDSs of the complete database) and against the corresponding species (read numbers were divided by the sum of the reads that mapped to the CDSs of the species). The coefficients of variation obtained for the three cheese replicates after normalization against the complete database were plotted as a function of the number of reads (Figure 3A). Overall, there was a low dispersion of the data for the cheese replicates. Indeed, the coefficients of variation were lower than 0.25 (25%) for most genes. The coefficients of variation were higher for the CDSs with low numbers of reads. This can be explained by the lower coverage of these CDSs by sequencing reads, resulting in more irregular results. In addition, we observed that the coefficients of variation regularly increased from day 5 to 35 (Figure 3B), which is probably the consequence of higher biological variations between the cheese replicates at the end of ripening. There was a very good correlation between the RNA-seq and the reverse transcription-quantitative PCR data. The correlation obtained for 80 *G. candidum* genes whose expression was previously measured by quantitative PCR in RNA samples in which rRNA was not depleted (Castellote et al., 2015) is shown in Figure 4. The *R*^2^ values corresponding to the linear regressions of the log_10_ fold changes were 0.811, 0.875, and 0.865 for day 14 vs. 5, day 19 vs. 5, and day 35 vs. 5 comparisons, respectively.

### Overview of the functional expression over time

**Figure 5:**
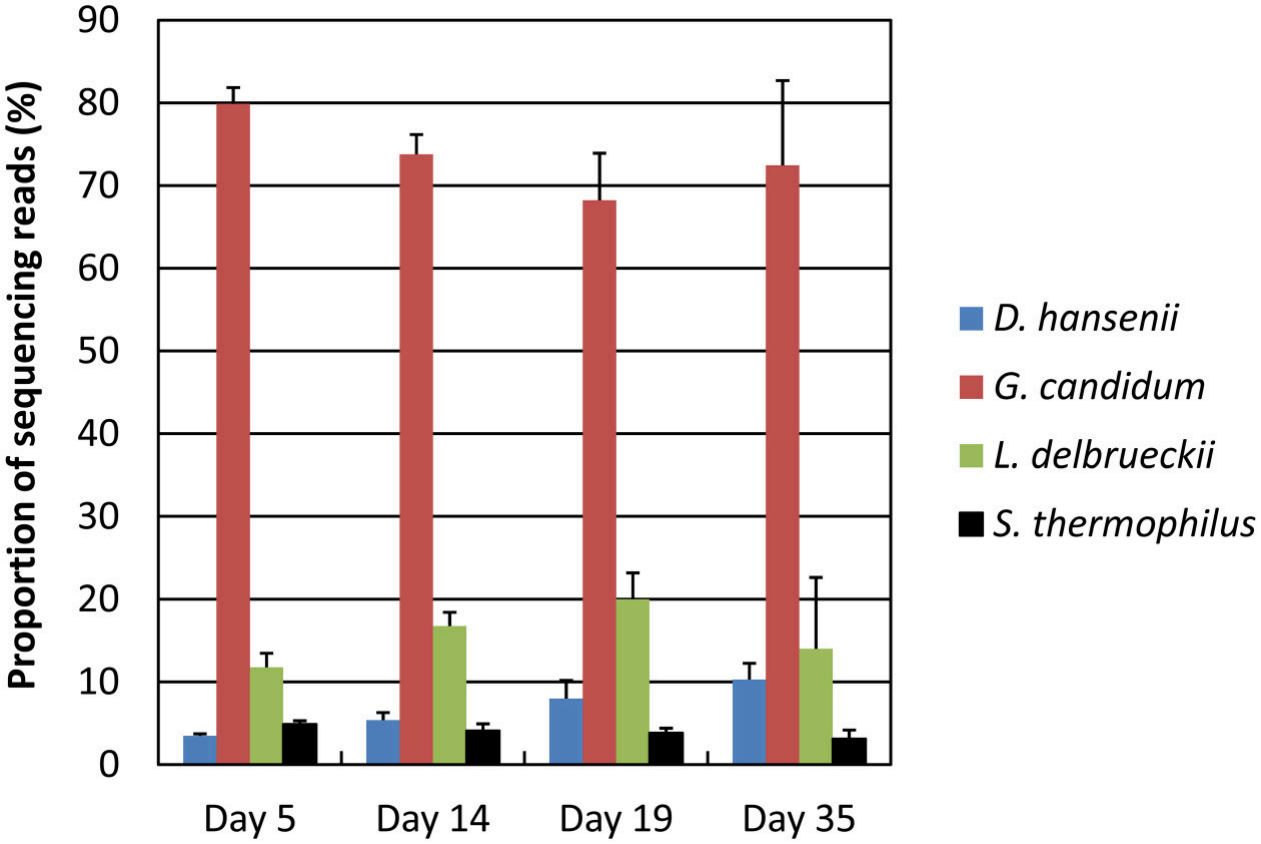
**Distribution of the sequencing reads at the four sampling days**. The RNA-seq data were normalized against the complete database. The upper error bars represent the standard deviations (three cheese replicates).

**Figure 6:**
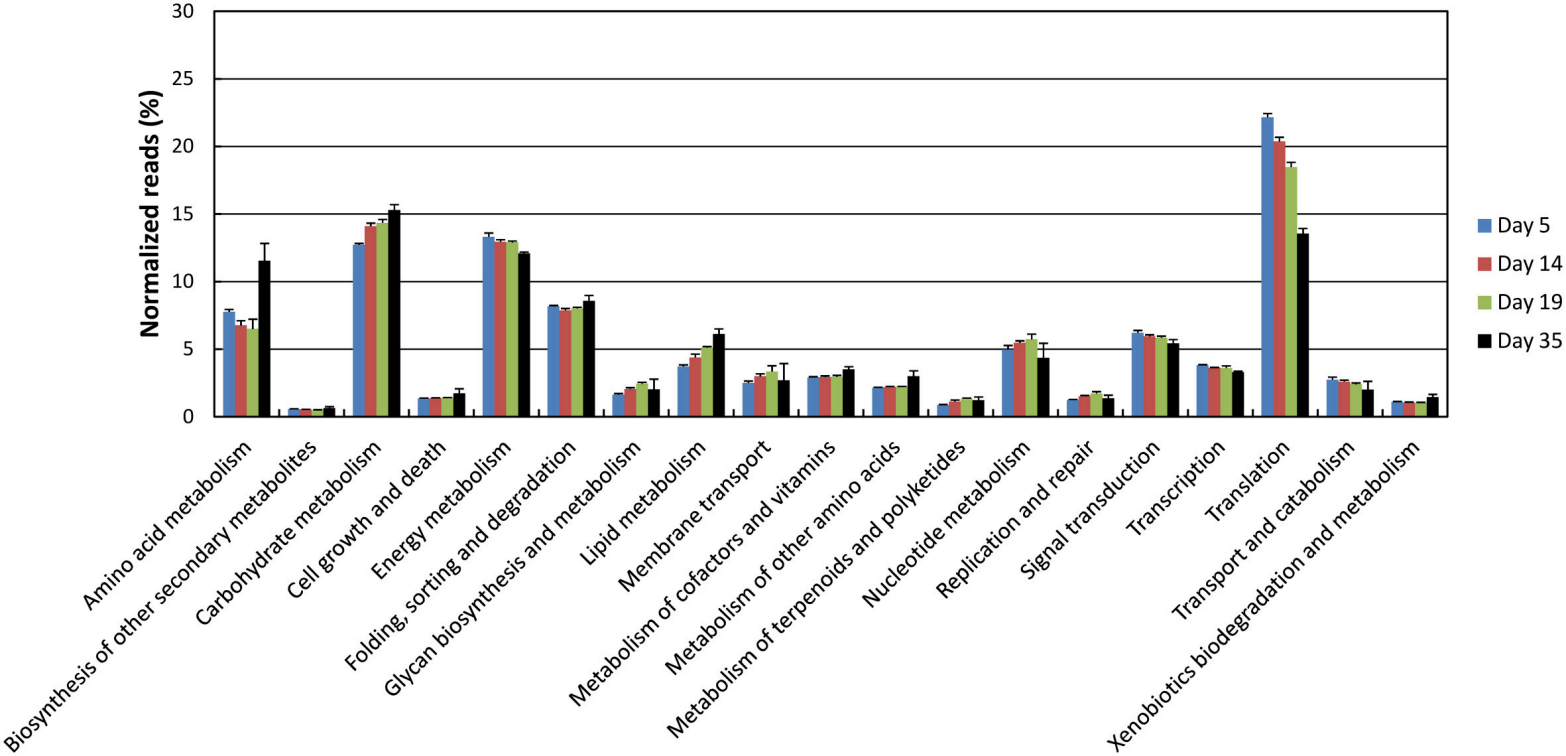
**Functional classification of the cheese rind metatranscriptome**. The functional classes were determined according to KEGG annotations of the CDSs. The number of reads was represented as a percentage of the total reads with KEGG assignments at each of the four sampling days, and the upper error bars represent the standard deviations (three cheese replicates).

Principal component analysis of the data normalized against the complete database showed a good clustering as a function of the sampling day (Supplementary Figure 1). Samples collected at days 14 and 19 were close together but separated from both the samples collected at day 5 and the samples collected at day 35. The proportion of *G. candidum* reads was quite stable during ripening, but increased for *D. hansenii* and decreased for *S. thermophilus* (Figure 5). It may be considered that these profiles are indicators of the relative activity of the strains during ripening. The global expression pattern during the ripening process was evaluated by classifying the sequencing reads according to KEGG annotations of the corresponding CDSs (Figure 6). The most abundant KEGG categories were Translation, Carbohydrate Metabolism, and Energy Metabolism. During ripening, the Carbohydrate Metabolism and Lipid Metabolism categories increased, and the Energy Metabolism and Translation categories decreased. In addition, the Amino Acid Metabolism category was higher at day 35 than at the other sampling times.

### Functional expression of the inoculated species

The transcriptomes generated after normalization of the RNA-seq data against the corresponding species were compared at the four sampling days. Principal component analysis of these transcriptomes showed a good clustering as a function of the sampling day for *G. candidum* and *D. hansenii* (Supplementary Figure 2). For these species, component one mainly separated the cheese rinds at day 5, 15, and 19, whereas component two mainly separated the cheese rinds at day 35 from the other samples. For *S. thermophilus* and *L. delbrueckii*, there was a larger dispersion of the cheese replicates, especially at day 35.

**Figure 7:**
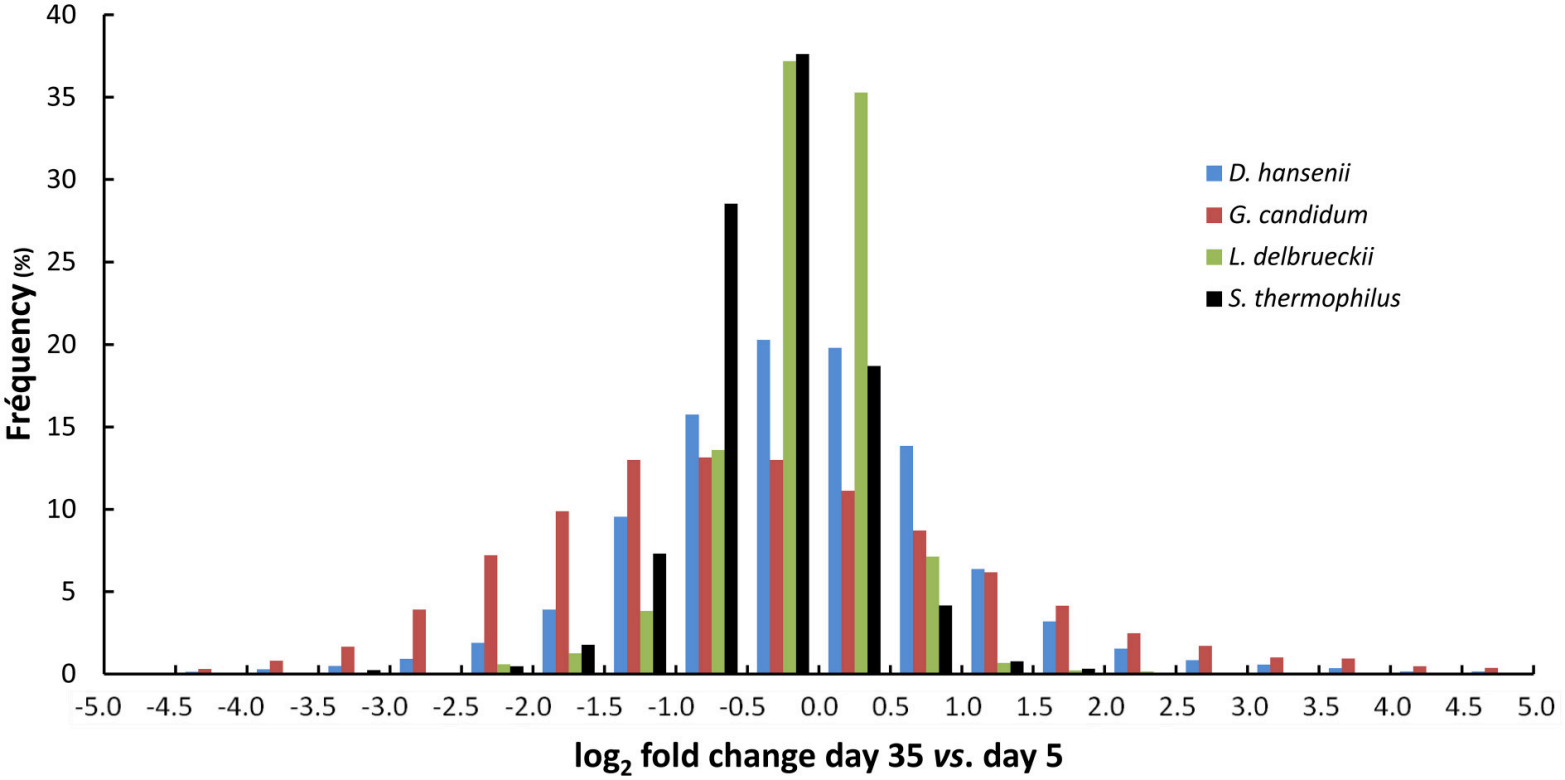
**Stability of the transcriptomes of *G. candidum*, *D. hansenii*, *L. delbrueckii*, and *S. thermophilus* during cheese ripening**. The diagram shows the distribution of the fold changes for the CDSs, expressed as log_2_ of fold change between day 5 and 35.

Comparison of the transcriptomes between day 5 and 35 showed that gene expression was more stable for the two lactic acid bacteria than for the two yeasts (Figure 7). When we considered the log_2_ of the fold change interval –1.0 to +1.0 (i.e., a fold change varying from 0.5 to 2), 89% of the *S. thermophilus* genes, and 93% of the *L. delbrueckii* genes were included in this interval, whereas this percentage decreased to 46 and 69% for *G. candidum* and *D. hansenii*, respectively. The higher stability of gene expression for the lactic acid bacteria was also confirmed by differential expression analysis (Table 1). Indeed, for all six comparisons (day 35 vs. 5, day 14 or 19, day 19 vs. 5 or 14, and day 14 vs. 5), the number of differentially expressed genes was lower for the lactic acid bacteria than for the yeasts. Between day 14 and 19, only 0.4% of their genes were differentially expressed.

**Table 1:** **Differentially expressed genes in the *G. candidum, D. hansenii, S. thermophiles*, and *L. delbrueckii* transcriptomes, expressed as percentages of the detected genes**.

	**Day 14**	**Day 19**	**Day 35**
***G. CANDIDUM***			
Day 5	59.5	68.5	84.2
Day 14		41.3	83.1
Day19			77.1
***D. HANSENII***			
Day 5	65.9	44.2	58.5
Day 14		9.2	55.6
Day19			50.9
***L. DELBRUECKII***			
Day 5	17.0	26.1	33.5
Day 14		0.4	25.8
Day19			1.7
***S. THERMOPHILUS***			
Day 5	19.2	29.1	30.5
Day 14		0.4	12.7
Day19			2.4

*Comparisons between the different sampling days were performed using the DESeq2 package*.

**Figure 8:**
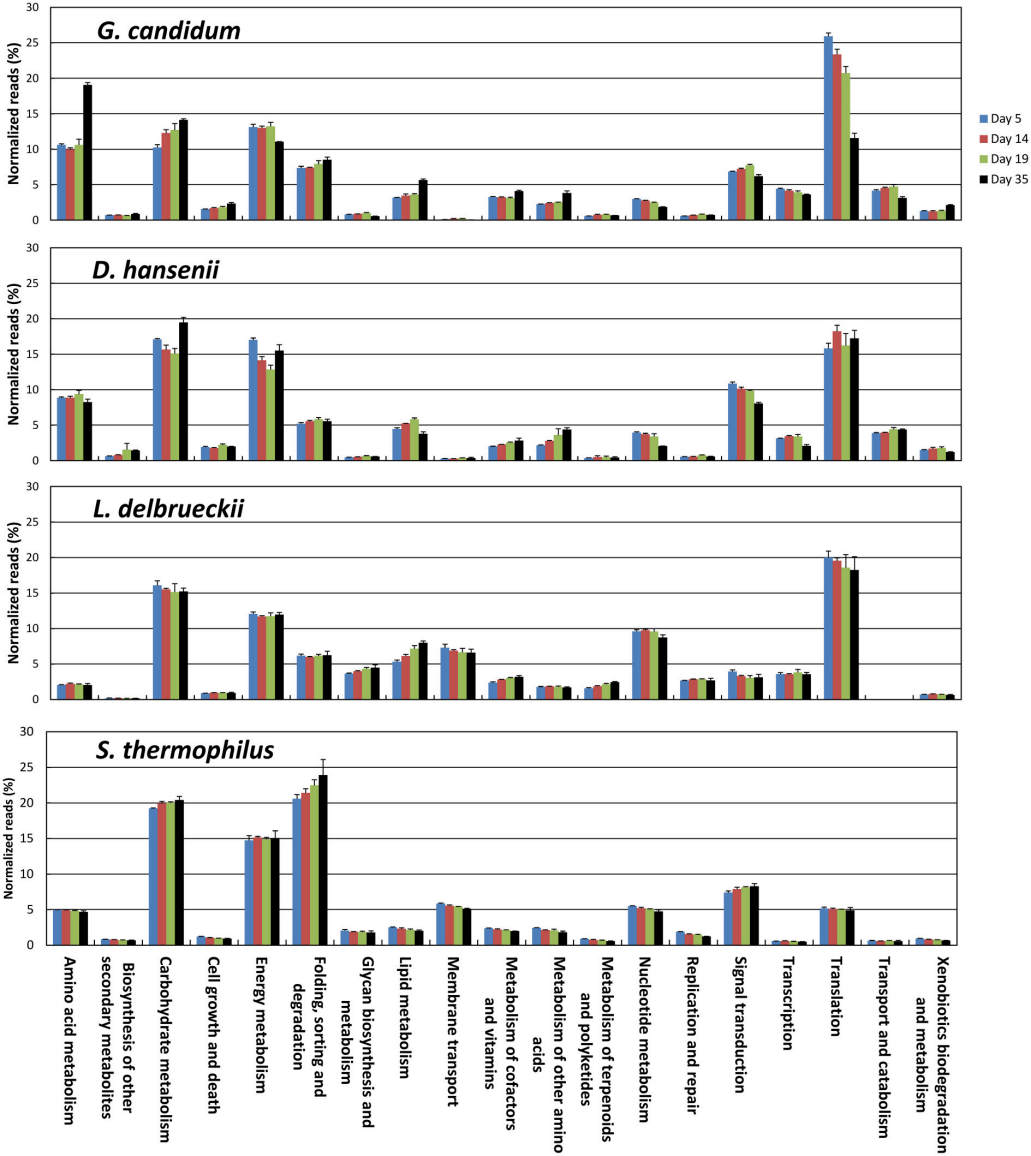
**Functional classification of the transcriptomes of *S. thermophilus*, *L. delbrueckii*, *D. hansenii*, and *G. candidum* in the cheese rinds**. The functional classes were determined according to KEGG annotations of the CDSs. The number of reads was represented as a percentage of the total reads of the species with KEGG assignments at each of the four sampling days, and the upper error bars represent the standard deviations (three cheese replicates).

The KEGG profiles obtained for the lactic acid bacteria were quite stable during ripening (Figure 8). The proportion of the categories Folding, Sorting, and Degradation, and Signal Transduction, was higher in the *S. thermophilus* transcriptome than in *L. delbrueckii*, whereas the opposite was observed for the categories Transcription, Translation, Nucleotide Metabolism, and Lipid Metabolism. The global distribution of the KEGG categories was quite similar in *G. candidum* and *D. hansenii*, However, for *G. candidum*, there was a large increase in the Amino Acid Metabolism category at day 35. For this yeast, there was also an increase in the Carbohydrate Metabolism and Lipid Metabolism categories, and a decrease in the Nucleotide Metabolism and Translation categories. Changes were also observed for *D. hansenii* during ripening, including an increase in the Metabolism of Other Amino Acids category and a decrease in the Nucleotide Metabolism category.

**Figure 9:**
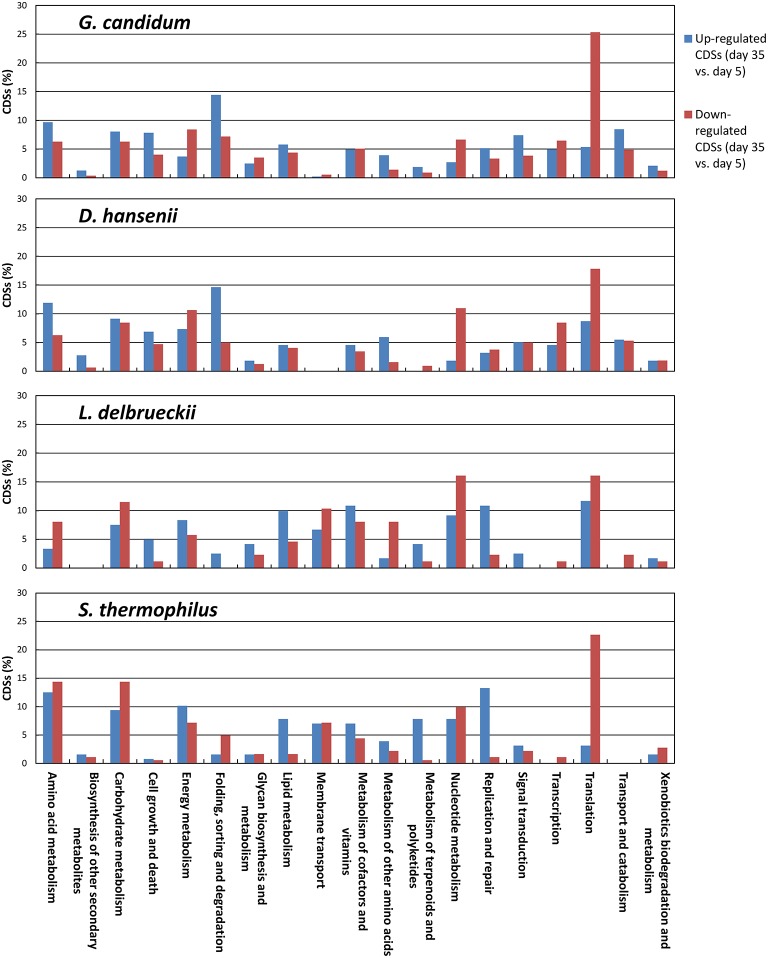
**Functional classification of the CDSs that were differentially expressed at day 35 in comparison to day 5**. Differential expression was evaluated using the DESeq2 package. For each species, results are represented as percentages relative to the sum of the KEGG entries generated from the up- or down-regulated genes.

In order to better characterize the functional changes in the transcriptomes during ripening, we determined the KEGG profiles of the CDSs for which an increase or a decrease was observed between day 5 and 35 (Figure 9). The number of KEGG entries generated from the CDSs that were differentially expressed was higher for the yeasts (540 and 1060 for *D. hansenii* and *G. candidum*, respectively) than for the lactic acid bacteria (353 and 226 for *S. thermophilus* and *L. delbrueckii*, respectively). For *D. hansenii*, the genes that were up-regulated at day 35 generated more KEGG entries in the Amino Acid Metabolism, Biosynthesis of Other Secondary Metabolites, Folding, Sorting and Degradation, and Metabolism of Other Amino Acids categories than the genes that were down-regulated. The opposite was observed for the Energy Metabolism, Nucleotide Metabolism, Transcription, and Translation categories. For *G. candidum*, the genes that were up-regulated at day 35 generated more KEGG entries in the Amino Acid Metabolism, Cell Growth and Death, Folding, Sorting and Degradation, Signal Transduction, and Transport and Catabolism categories. The opposite was observed for the Energy Metabolism, Nucleotide Metabolism, and Translation categories. Overall, the KEGG functional profiles of the differentially expressed genes were quite similar in *G. candidum* and *D. hansenii*, but differences between the up- and the down-regulated genes were more pronounced for the Nucleotide Metabolism category in *D. hansenii*, and for the Translation category in *G. candidum*. In *L. delbrueckii*, more genes were up-regulated than down-regulated in several categories, especially Lipid Metabolism and Replication and Repair. The categories in which more genes were down-regulated than up-regulated include the Amino Acid Metabolism, Metabolism of Other Amino Acids, and Nucleotide Metabolism categories. For *S. thermophilus*, more genes in the Translation category were down-regulated than up-regulated at the end of ripening. This change was greater than in *L. delbrueckii*.

The availability of the transcriptomes of the two lactic acid bacteria and the two yeasts at the four sampling times provides new insights into many aspects of the physiology of these strains during cheese ripening (Supplementary File 1). Hereafter, we present selected results concerning *G. candidum* and *D. hansenii*, which are summarized in Supplementary Table 2.

Expression of the four *G. candidum* and the three *D. hansenii* genes encoding ammonium importers strongly decreased during cheese ripening, which probably reveals the presence of excess ammonia in the cells at the end of ripening. *G. candidum* had two paralogs of the ammonium exporter ATO2, one of which was up-regulated at the end of ripening, whereas the other one was down-regulated. The same result was observed for *D. hansenii*. Ammonia can be assimilated by the glutamate synthase cycle (GLT1/GLN1). In *G. candidum*, there was a down-regulation of GLT1 and of one of the two GLN1 genes at day 14 and day 19. In *D. hansenii*, there was a strong decrease of GLT1 expression during ripening, which may reveal a decrease of ammonia assimilation through this pathway. In both yeasts, there was also a decrease in the expression of assimilatory NADP-dependent glutamate dehydrogenase genes (GDH1 or GDH3). NAD^+^-dependent glutamate dehydrogenase (GDH2) degrades glutamate to ammonia and alpha-ketoglutarate. In *G. candidum*, expression of the two GDH2 paralogs increased from day 5 to 35, whereas in *D. hansenii*, GDH2 was up-regulated only at day 35. Glutamate may also be degraded to succinate, via 4-aminobutyrate, through the combined action of GAD1, UGA1, and UGA2. In *G. candidum*, expression of GAD1, of one of the two UGA1 paralogs and of the two UGA2 paralogs increased at the end of ripening, which may indicate that glutamate was increasingly degraded through this pathway. In addition, there was a strong down-regulation of the 4-aminobutyrate importers UGA4 in both yeasts, possibly revealing an excess of intracellular 4-aminobutyrate. In *G. candidum*, expression of four of the five genes and paralogs involved in degradation of arginine to proline increased at the end of ripening, whereas in *D. hansenii*, these genes were mostly down-regulated. In addition, in *G. candidum*, PUT1, and PUT2, which are involved in the degradation of proline to glutamate, were more expressed from day 14 to 35 than at day 5, whereas the opposite was observed in *D. hansenii*. In the two yeasts, expression of the GCV1 and GCV2 genes involved in glycine catabolism was higher at day 35 than at day 5, but in *D. hansenii*, these genes were also down-regulated at day 14 and 19. A similar pattern was observed for CHA1, whose product (serine/threonine dehydratase) catalyzes the degradation of serine into pyruvate and of threonine into alpha-ketobutyrate. Nine and seven genes are annotated as amino-acid transaminases in the *G. candidum* and *D. hansenii* genomes, respectively. Amino acid transaminases catalyze the first step of several amino acid degradation pathways. No uniform expression profile was observed for these genes. However, when we considered the sum of the sequencing reads that mapped to these genes, which can be considered as an indicator of their global expression level, a higher value was observed at day 35 than at day 5 for both yeasts, suggesting that the yeasts actively degraded amino acids at the end of ripening. Different expression profiles were observed for the amino acid permease genes, and no major change in their global expression level was observed during ripening in either of the two yeasts.

Lipolysis of the cheeses increased during ripening (Supplementary Table 3), which means that increasing amounts of free fatty acids were available as an energy source for the cheese microorganisms. In *D. hansenii* and *G. candidum*, no uniform expression profile was observed for the genes involved in fatty acid catabolism. However, the global expression level of these genes was slightly lower at day 35 than at the earlier sampling times. The cells therefore do not seem to enhance the catabolism of fatty acids at the end of ripening.

Expression of the *D. hansenii* genes, LAC4, and LAC12, involved in lactose import and its conversion to galactose and glucose, was higher at day 14 and 19 than at the other sampling days. However, lactose was nearly exhausted at day 5 (Supplementary Table 3), and it therefore does not constitute a major energy substrate during the ripening period investigated in the present study. Concerning the catabolism of galactose, the global expression level of the corresponding genes in *G. candidum* and *D. hansenii* (GAL10, GAL1, GAL7, and PGM2) was lower from day 14 to 35 than at day 5. This compound was exhausted from cheese after ~19 days of ripening (Supplementary Table 3).

Most lactate was exhausted at day 35 (Supplementary Table 3; Castellote et al., 2015). In contrast to expectations, the global expression level of genes possibly involved in its catabolism (lactate transporters and lactate dehydrogenases) was not lower at this sampling time than at earlier sampling times when this substrate was more abundant.

In general, expression of genes encoding the subunits of the electron transport chain complexes (NADH:ubiquinone oxidoreductase, succinate dehydrogenase (ubiquinone), ubiquinol cytochrome c oxidoreductase, and cytochrome c oxidase) decreased in the two yeasts during ripening. Expression of the F1Fo ATP synthase subunit genes was quite stable in *D. hansenii*, whereas a strong decrease was observed at day 35 in *G. candidum*.

In *D. hansenii*, most genes involved in iron acquisition had similar expression profiles, suggesting a common regulation. The global expression level of these genes was higher from day 14 to 35 than at day 5. This was not observed in *G. candidum*, in which these genes were globally less expressed at day 35.

In *G. candidum*, expression of the genes encoding the two first steps of NAD *de novo* biosynthesis (i.e., BNA2 and BNA7) increased during ripening. Even if this increase was not observed for the other genes of this pathway, the global expression of the genes was much higher at day 35 than at the other sampling days. No increase was observed for the *D. hansenii* BNA2 gene (BNA7 was not present in its genome annotation).

At day 35, the *D. hansenii* carbonic anhydrase gene (NCE103) and one of the two *G. candidum* paralogs were strongly down-regulated in comparison to day 5, which is probably the consequence of a higher availability in carbon dioxide. Expression of PMA1, encoding a plasma membrane H^+^-ATPase, the major regulator of cytoplasmic pH, decreased during ripening, which may be the consequence of the cheese curd de-acidification. This decrease was higher in *G. candidum* than in *D. hansenii*.

## Discussion

In the present study, we investigated the activity of the microorganisms inoculated in a Reblochon-style cheese using high-throughput RNA sequencing. A large number of CDS reads were generated for four of the five species that were inoculated into the cheese, which enabled us to study their individual transcriptomes. For the fifth species, *B. aurantiacum*, only 2% of the CDSs were detected, which was due to poor growth. Many cases of failure of commercial strains to establish themselves on the surface of cheeses have been reported in the past (Brennan et al., 2002; Feurer et al., 2004; Mounier et al., 2005; Goerges et al., 2008), emphasizing the importance of careful selection of cheese-ripening cultures. Our transcriptomic analyses involved depletion of rRNA from the sample and short read sequencing followed by mapping against reference genomes. This resulted in a higher sensitivity in comparison to the two cheese metratranscriptomic studies that were previously published (Lessard et al., 2014; Dugat-Bony et al., 2015). In addition, the metatranscriptomes generated from cheese replicates were very similar, showing the high homogeneity of the cheese manufacturing process and the good reliability of the transcriptomic analysis procedure. This latter point was corroborated by the excellent correlation obtained with quantitative PCR measurements.

The rRNA depletion procedure that we used involved the simultaneous depletion of prokaryotic and eukaryotic rRNA using Ribo-Zero kits, which are based on the capture of rRNA by rRNA-specific probes. This method was also used by Hesse et al. (2015) for the metatranscriptomic analysis of forest floor communities, resulting in the presence of 31 to 51% rRNA in the sequencing reads. These percentages are quite similar to those obtained for the cheeses investigated in the present study. Indeed, in our datasets, the proportion of rRNA reads was about 20% from day 5 to 19, and increased to about 50% at day 35. This presence of rRNA may be explained by to the fact that some rRNA, such as 5S rRNA and mitochondrial rRNA, are frequently not removed by the Ribo-Zero kits (http://www.illumina.com/products/rrna-removal-kit-species-compatibility.html). The increase in the proportion of rRNA reads at day 35 may be explained by a lower rRNA integrity. However, another explanation is the fact that the global transcriptional activity of the microorganisms is probably lower at the end of ripening, resulting in a lower amount of mRNA in comparison to the rRNAs that are not depleted by the Ribo-Zero kit. A similar phenomenon may also contribute to increasing the proportion of reads that did not map to the database at day 35. Indeed, in addition to the fact that these reads include CDS reads from the adventitious microorganisms that grow at the end of ripening, they may also correspond to non-coding RNA (such as regulatory RNA, or tRNA and rRNA from adventitious microorganisms, whose sequences are not in the database) and whose proportion would be higher when the transcriptional activity decreases. The presence of rRNA reads has no influence on the metatranscriptome datasets since these datasets are only composed of CDS reads. However, a poor removal of rRNA would result in a low number of CDS reads and would consequently decrease the detection of these CDSs and increase the variability of the quantifications due to insufficient CDS coverage by the reads.

One way to investigate metatranscriptomic datasets is to consider the microbiota as a whole and to investigate the composition and changes of the metatranscriptomes in terms of function or of phylogenetic assignation. This is typically performed after normalization of the RNA-seq data against the complete database (e.g., by dividing read numbers for each CDS by the sum of the reads that mapped to the CDSs from all the reference genomes). Using this approach, we observed that a large proportion of the detected transcripts corresponded to genes involved in translation, but this proportion decreased with the ripening time, suggesting that the microbiota globally decreased its translational activity. We also observed that there was a higher abundance of transcripts involved in amino acid metabolism at the end of ripening, which most probably indicates an increase in the degradation of amino acids originating from caseins. Such analyses also revealed that *G. candidum* accounted for the largest proportion of the CDS reads at all ripening times, reflecting the importance of this species for the activity of the cheese rind microbiota. The global abundance of *D. hansenii* genes increased during ripening, suggesting that *D. hansenii* cells became more abundant and/or more active within the microbiota. The opposite was observed for the lactic acid bacterium *S. thermophilus*. However, it must be kept in mind that the RNA-seq data do not necessarily reflect the true abundance of the transcripts from the different species within the microbiota due to possible differences in RNA extraction efficiency between the species. In addition, the transcription and translation machineries from fungi are different from that of bacteria, e.g., in terms of RNA stability. This is why caution must be taken when analyzing functional classes that are built from the combination of bacterial and fungal sequencing reads. It must also be kept in mind that the metatranscriptomes generated here do not include the contribution of adventitious microorganisms such as strains related to the *L. casei* group, whose genomes were not included in the reference database.

The individual transcriptomes of *S. thermophilus, L. delbrueckii, D. hansenii*, and *G. candidum* were generated after normalization of the RNA-seq data against the corresponding species (read numbers for each CDS were divided by the sum of the reads that mapped to the CDSs of the species). The main advantage of this type of normalization is that it can be used to detect and quantify up- or down-regulations of individual genes in the different samples for each species, regardless of differences in cell biomass, in RNA extraction efficiency, or in rRNA removal efficiency. This provides useful insights into the physiological changes that occur during cheese ripening, such as changes in energy substrates, anabolic reactions, or stresses. Analysis of the distribution of the fold change values of the CDSs revealed that only minor changes occurred in the transcriptomes of the two lactic acid bacteria. This may be explained by the fact that lactic acid bacteria from starter cultures grow at the very beginning of cheesemaking and probably have a limited activity during the ripening period that was investigated here (day 5–35). However, for the KEGG functional classes, Lipid Metabolism, and Replication and Repair, we observed that more genes were up-regulated than down-regulated at day 35. Considerable changes were observed in the transcriptomes of the yeasts during ripening. For example, a significant proportion of fungal genes had fold change values between day 5 and 35 higher than 4 or lower than 0.25 (corresponding to log_2_ of fold change >2 or < –2, respectively). In addition, similar profiles of up- or down-regulations were observed for several genes belonging to the same function, probably as a result of co-regulations. This allows us to better assess the changes that occur in the cells during ripening.

Biochemical analyses of the cheeses showed that only a low amount of lactose was present at day 5, and that most of the galactose and lactate were exhausted at day 19 and 35, respectively. In addition, there was a large increase of ammonia from day 19 to 35. This suggested a transition from the catabolism of galactose and lactate toward the catabolism of amino acids during ripening. Investigation of the changes in the transcriptomes of the yeasts enabled us to better characterize this transition. Most ammonium importers were strongly down-regulated after day 5, indicating that this compound had become more abundant in the cells, possibly as a consequence of higher amino acid availability. Genes involved in the assimilation of ammonia through the NADP-dependent glutamate dehydrogenase or the glutamate synthase cycle were also down-regulated, showing that less glutamate was synthesized *de novo* by the cells. The main biological function of the aerobic catabolism of amino acids by cheese microorganisms is to produce energy. The NAD-dependent glutamate dehydrogenase catalyzes the main pathway for glutamate catabolism in yeasts (Miller and Magasanik, 1990), yielding NADH, ammonia, and alpha-ketoglutarate, which can enter into the TCA cycle. The abundance of *G. candidum* NAD-dependent glutamate dehydrogenase gene transcripts strongly increased during ripening, whereas in *D. hansenii*, the abundance decreased at day 14 and 19, and increased afterwards. Similar behavior was observed for the genes involved in the catabolism of proline to glutamate, of glycine, of serine, and of threonine. This suggests a similar regulation for the catabolism of these amino acids, as well as the fact that they are consumed later in *D. hansenii* than in *G candidum*. Another difference between the two yeasts is that the expression of genes involved in the catabolism of arginine increased in *G. candidum*, whereas it decreased in *D. hansenii*, which may reveal a higher catabolism of this amino acid by *G. candidum* at the end of ripening. In both yeasts, the global expression level of amino acid transaminases was higher at day 35, which is in accordance with the fact that they seem to catabolize more amino acids at the end of ripening. Concerning the free fatty acids, we observed that even if their amount increased throughout cheese ripening, the cells did not seem to up-regulate the genes involved in their catabolism at the end of ripening.

Galactose is an energy substrate for *G. candidum* and *D. hansenii* (Boutrou and Gueguen, 2005; Breuer and Harms, 2006). In Reblochon-type cheeses, it is produced from lactose by the lactic acid bacteria at the beginning of cheesemaking. Interestingly, the transcript abundances for the genes involved in galactose catabolism decreased in both yeasts, which is probably a consequence of the exhaustion of this substrate during ripening. However, although almost all lactate was exhausted at the end of ripening, the genes involved in its catabolism were not down-regulated at day 35. This suggests that there is no transcriptional regulation of lactate catabolism by lactate availability in these yeasts.

Ammonia production by the yeasts significantly contributes to increasing the pH of the cheese rind. Indeed, its production represents 87 mmol/kg of cheese at day 35, which is not far from the amount of lactate consumed from day 5 to 35 (101 mmol/kg). Ammonia is not considered to be toxic for bacteria (Müller et al., 2006). However, it has been shown that it is sometimes toxic in yeasts, e.g., at low K^+^ concentration or when ammonia importers are over-expressed (Hess et al., 2006). To our knowledge, there is no study devoted to the possible toxic effect of ammonia when it is produced inside the cells, as is the case for *G. candidum*, and in *D. hansenii* when they catabolize amino acids. It can be hypothesized that if there is a toxic effect due to high internal accumulation, this would result in a decreased cellular activity at the end of ripening, especially for *G. candidum*, in which the catabolism of amino acids seems to be induced earlier than in *D. hansenii*. Interestingly, for both yeasts, there was a decrease in the abundance of gene transcripts from electron transport chain complexes, which suggests a lower cellular activity. In addition, for *G. candidum*, there was a down-regulation of the genes encoding F1F0 ATP synthase subunits, which probably reflects decreased production and consumption of ATP.

In this study, other interesting changes during ripening were pointed out, such as down-regulation of the plasma membrane H^+^-ATPase at day 35, which was probably caused by the increase in the cheese pH, and up-regulation of iron acquisition genes by *D. hansenii*, which suggests limited iron availability for this species, as already observed for several cheese-ripening microorganisms (Monnet et al., 2012). The transcriptomic data generated in the present study can thus be used to investigate various other metabolisms or physiological features of *G. candidum* and *D. hansenii* in cheese. However, for many genes that were differentially expressed, none or limited functional annotations were available. Indeed, the *G. candidum* ATCC 204307 genome contains 1245 genes encoding conserved hypothetical proteins with no ortholog in *Saccharomyces cerevisiae*, and 846 genes with no similarity to any gene outside *G. candidum* (Morel et al., 2015). A better functional characterization of these species would thus help to take better advantage of the transcriptomic data.

Metatranscriptomic analyses may help to better control cheesemaking processes. For example, improved knowledge of the microbial physiology at different stages of the ripening process will help to better understand the impact of the different species of the microbiota on the cheese matrix. In addition, the occurrence of manufacturing defects can be studied by comparing problematic manufacturing runs with standard runs. Metatranscriptomic analyses can also be used to identify real-time PCR bioindicators relevant for cheesemaking, as proposed by Dugat-Bony et al. (2015). When transcript abundances measured by real-time PCR are normalized against stable reference genes, the resulting bioindicators characterize the cellular activity or physiological state. However, abundances may also be standardized against the amount of cheese (Monnet et al., 2013; Castellote et al., 2015). In that case, the bioindicators reflect the overall abundance of transcripts in the cheese. This is very useful for monitoring the expression of genes encoding enzymes that catalyze key biochemical reactions in cheeses, such as the NAD-dependent glutamate dehydrogenase. Indeed, this enzyme is responsible for the extensive production of ammonia in cheese, and generates alpha-ketoglutarate, which stimulates the production of aroma compounds from various amino acids (Tanous et al., 2002).

In conclusion, the present study shows that it is possible to generate cheese metatranscriptomes that are appropriate for creating a global picture of cellular function in each of the dominant species. This is a powerful tool for understanding the growth and activity of cheese-ripening microorganisms. In the cheeses that we investigated, we were able to identify transcriptional regulations concerning the catabolism of energy substrates by *G. candidum* and *D. hansenii*. At the end of ripening, we also observed a down-regulation of electron transport chain components, suggesting a lower cellular activity that probably has multifactorial causes, one of which could be the high production of ammonia from amino acids. It should be noted that the transcriptomic data investigated here were obtained during one cheesemaking run. Even if they provide interesting information about the physiological changes that occur in these cheeses, the activity of the microorganisms may also be affected by factors such as the composition of the cheese milk, whose effect was not studied here.

## Author contributions

Analyzed the transcriptomic data: CM, DS, JB, FI, PB. Coordinated the study and wrote the paper: CM. Planned the cheese manufacturing runs and analyses: SF. Performed the 16S metabarcoding experiments and bioinformatic analyses: ED.

### Conflict of interest statement

The authors declare that the research was conducted in the absence of any commercial or financial relationships that could be construed as a potential conflict of interest.
