# Supplementary material for: Investigation of the Activity of the Microorganisms in a Reblochon-Style Cheese by Metatranscriptomic Analysis
Source: Front Microbiol. 2016 Apr 20;7:536. doi: 10.3389/fmicb.2016.00536 (PMC4837152; doi:10.3389/fmicb.2016.00536)
Supplement: Supplementary file 1 [file Table1.PDF]

Supplementary Table 1. Bacterial community structure of the cheese rinds as determined by 16S rRNA gene metabarcoding analysis. One cheese sample was analysed at each sampling day.

	**OTU proportion (%)**				
**Species**	**Day1**	**Day5**	**Day14**	**Day19**	**Day35**
*Streptococcus thermophilus**	65.536	80.906	72.900	77.731	75.780
*Lactobacillus delbrueckii**	34.403	17.833	21.002	17.575	11.029
Lactobacillus group casei	0	0.013	4.461	2.985	9.179
Weissella hellenica	0	1.070	0.056	0	0
Lactobacillus parabuchneri	0	0	0.258	0.670	0.810
Pseudomonas putida	0	0	0.247	0.421	0.273
*Brevibacterium aurantiacum**	0.004	0.004	0	0	0.901
Enterobacter kobei	0.004	0.013	0.311	0.182	0.024
Lactococcus lactis	0	0.030	0.322	0.056	0.043
Corynebacterium variabile	0	0.021	0.016	0.019	0.369
Arthrobacter arilaitensis	0	0	0	0.026	0.388
Brachybacterium tyrofermentans	0	0	0	0.007	0.331

* species that were inoculated in the cheeses
