# Supplementary material for: Investigation of the Activity of the Microorganisms in a Reblochon-Style Cheese by Metatranscriptomic Analysis
Source: Front Microbiol. 2016 Apr 20;7:536. doi: 10.3389/fmicb.2016.00536 (PMC4837152; doi:10.3389/fmicb.2016.00536)
Supplement: Supplementary file 2 [file Table2.PDF]

Supplementary Table 2. Expression levels of selected genes from G. candidum and D. hansenii. Fold changes were calculated after normalization against the corresponding species (sum of the reads that mapped on the CDSs of the species) and are relative to day 5 (white=no change; red=down-regulation; green=up-regulation)
Differentially expressed genes between day 5 and day 35 are indicated by an asterisk (DESeq2 package, adjusted p-value<0.05)

			Log2 of fold change vs. day 5:				
**Species**	**CDS locus tag**	**CDS product**	**Day 5**	**Day 14**	**Day 19**	**Day 35**	
**Ammonium importers:**							
G. candidum	GECA02s04113g	similar to Saccharomyces cerevisiae YGR121C MEP1 Ammonium permease, belongs to a ubiquitous family of cytoplasmic membrane proteins that transport only ammonium	0.00	-5.06	-5.67	-5.84	*
G. candidum	GECA12s00934g	similar to Saccharomyces cerevisiae YNL142W MEP2 Ammonium permease involved in regulation of pseudohyphal growth	0.00	-6.46	-7.97	-5.01	*
G. candidum	GECA07s05499g	similar to Saccharomyces cerevisiae YNL142W MEP2 Ammonium permease involved in regulation of pseudohyphal growth	0.00	-0.31	-0.24	-1.96	*
G. candidum	GECA17s01935g	similar to Saccharomyces cerevisiae YPR138C MEP3 Ammonium permease of high capacity and low affinity	0.00	-2.26	-3.49	-4.04	*
D. hansenii	DEHA2G02156g	similar to Saccharomyces cerevisiae YGR121C MEP1 Ammonium permease, belongs to a ubiquitous family of cytoplasmic membrane proteins that transport only ammonium	0.00	-0.59	-1.21	-4.54	*
D. hansenii	DEHA2D03234g	similar to Saccharomyces cerevisiae YNL142W MEP2 Ammonium permease involved in regulation of pseudohyphal growth	0.00	-5.73	-7.87	-8.59	*
D. hansenii	DEHA2F24442g	similar to Saccharomyces cerevisiae YPR138C MEP3 Ammonium permease of high capacity and low affinity	0.00	0.19	0.11	-1.10	*
**Ammonium exporters:**							
G. candidum	GECA07s02331g	similar to Saccharomyces cerevisiae YNR002C ATO2 Putative transmembrane protein involved in export of ammonia	0.00	-1.04	1.64	4.07	*
G. candidum	GECA18s01352g	similar to Saccharomyces cerevisiae YNR002C ATO2 Putative transmembrane protein involved in export of ammonia	0.00	0.35	-0.22	-0.93	*
D. hansenii	DEHA2F17996g	similar to Saccharomyces cerevisiae YNR002C ATO2 Putative transmembrane protein involved in export of ammonia	0.00	-0.01	0.26	2.40	*
D. hansenii	DEHA2C15158g	similar to Saccharomyces cerevisiae YNR002C ATO2 Putative transmembrane protein involved in export of ammonia	0.00	0.52	0.21	-3.87	*
**Glutamate synthase cycle:**							
G. candidum	GECA06s01880g	similar to Saccharomyces cerevisiae YDL171C GLT1 NAD(+)-dependent glutamate synthase (GOGAT)	0.00	-0.34	-0.62	0.75	*
G. candidum	GECA32s02430g	similar to Saccharomyces cerevisiae YPR035W GLN1 Glutamine synthetase (GS)_ synthesizes glutamine from glutamate and ammonia	0.00	-2.24	-3.25	0.65	*
G. candidum	GECA13s03288g	similar to Saccharomyces cerevisiae YPR035W GLN1 Glutamine synthetase (GS)_ synthesizes glutamine from glutamate and ammonia	0.00	-0.11	0.53	1.16	*
D. hansenii	DEHA2G15642g	uniprot|Q5EN26 Debaryomyces hansenii GLT1 Glutamate synthase	0.00	-3.01	-3.52	-4.19	*
D. hansenii	DEHA2G19140g	similar to Saccharomyces cerevisiae YPR035W GLN1 Glutamine synthetase (GS)_ synthesizes glutamine from glutamate and ammonia	0.00	-0.08	-0.11	-0.16	
**NADP-dependent glutamate dehydrogenase (glutamate synthesis)**:							
G. candidum	GECA13s00659g	similar to Saccharomyces cerevisiae YAL062W GDH3 NADP(+)-dependent glutamate dehydrogenase	0.00	-1.64	-2.17	-1.17	*
D. hansenii	DEHA2C17204g	uniprot|Q9HGS2 Debaryomyces hansenii GDH1 NADP- dependent glutamate dehydrogenase	0.00	-0.27	-0.56	-2.76	*
**NAD-dependent glutamate dehydrogenase (glutamate catabolism):**							
G. candidum	GECA06s00802g	similar to Saccharomyces cerevisiae YDL215C GDH2 NAD(+)-dependent glutamate dehydrogenase	0.00	2.54	3.25	4.00	*
G. candidum	GECA05s06082g	similar to Saccharomyces cerevisiae YDL215C GDH2 NAD(+)-dependent glutamate dehydrogenase	0.00	0.01	0.98	1.03	*
D. hansenii	DEHA2C10054g	similar to Saccharomyces cerevisiae YDL215C GDH2 NAD(+)-dependent glutamate dehydrogenase	0.00	-1.20	-0.67	1.70	*
**Catabolism or glutamate to succinate:**							
G. candidum	GECA08s02727g	similar to Saccharomyces cerevisiae YMR250W GAD1 Glutamate decarboxylase, converts glutamate into gamma-aminobutyric acid (GABA) during glutamate catabolism	0.00	0.02	0.48	2.22	*
G. candidum	GECA06s01990g	similar to Saccharomyces cerevisiae YGR019W UGA1 Gamma-aminobutyrate (GABA) transaminase involved in the 4-aminobutyrate and glutamate degradation pathways	0.00	1.17	2.17	3.38	*
G. candidum	GECA09s02386g	similar to Saccharomyces cerevisiae YGR019W UGA1 Gamma-aminobutyrate (GABA) transaminase involved in the 4-aminobutyrate and glutamate degradation pathways	0.00	-0.12	0.59	-0.05	*
G. candidum	GECA15s02936g	similar to Saccharomyces cerevisiae YBR006W UGA2 Succinate semialdehyde dehydrogenase involved in the utilization of gamma-aminobutyrate (GABA)	0.00	0.27	1.13	1.72	*
G. candidum	GECA01s11296g	similar to Saccharomyces cerevisiae YBR006W UGA2 Succinate semialdehyde dehydrogenase involved in the utilization of gamma-aminobutyrate (GABA)	0.00	0.85	1.36	1.07	*
G. candidum	GECA10s00989g	similar to Saccharomyces cerevisiae YDL210W UGA4 Permease that serves as a gamma-aminobutyrate (GABA) transport protein involved in the utilization of GABA	0.00	-0.96	-0.62	-3.79	*
G. candidum	GECA06s02177g	similar to Saccharomyces cerevisiae YDL210W UGA4 Permease that serves as a gamma-aminobutyrate (GABA) transport protein involved in the utilization of GABA	0.00	-0.65	-0.97	-0.35	

D. hansenii	DEHA2F10450g	similar to Saccharomyces cerevisiae YMR250W GAD1 Glutamate decarboxylase, converts glutamate into gamma-aminobutyric acid (GABA) during glutamate catabolism	0.00	0.55	0.44	0.14	
D. hansenii	DEHA2C16324g	similar to Saccharomyces cerevisiae YGR019W UGA1 Gamma-aminobutyrate (GABA) transaminase involved in the 4-aminobutyrate and glutamate degradation pathways	0.00	-0.24	1.20	0.96	*
D. hansenii	DEHA2F09306g	similar to Saccharomyces cerevisiae YGR019W UGA1 Gamma-aminobutyrate (GABA) transaminase involved in the 4-aminobutyrate and glutamate degradation pathways	0.00	-2.67	-1.90	-2.15	*
D. hansenii	DEHA2B10384g	similar to Saccharomyces cerevisiae YBR006W UGA2 Succinate semialdehyde dehydrogenase involved in the utilization of gamma-aminobutyrate (GABA)	0.00	0.01	0.84	0.03	
D. hansenii	DEHA2B06556g	similar to Saccharomyces cerevisiae YBR006W UGA2 Succinate semialdehyde dehydrogenase involved in the utilization of gamma-aminobutyrate (GABA)	0.00	0.19	0.03	-0.33	
D. hansenii	DEHA2E14696g	weakly similar to Saccharomyces cerevisiae YBR006W UGA2 Succinate semialdehyde dehydrogenase involved in the utilization of gamma-aminobutyrate (GABA)	0.00	-0.09	-0.16	-1.30	*
D. hansenii	DEHA2G22330g	similar to Saccharomyces cerevisiae YDL210W UGA4 Permease that serves as a gamma-aminobutyrate (GABA) transport protein involved in the utilization of GABA	0.00	-3.99	-4.45	-3.58	*
D. hansenii	DEHA2D18964g	similar to CA3801|CaUGA4 Candida albicans CaUGA4 GABA-specific transport protein	0.00	-1.89	-1.82	-2.95	*
**Catabolism of arginine to proline:**							
G. candidum	GECA06s03409g	similar to Saccharomyces cerevisiae YPL111W CAR1 Arginase	0.00	0.75	1.52	3.37	*
G. candidum	GECA18s02397g	similar to Saccharomyces cerevisiae YPL111W CAR1 Arginase	0.00	1.12	2.52	2.87	*
G. candidum	GECA01s11263g	similar to Saccharomyces cerevisiae YPL111W CAR1 Arginase	0.00	-0.13	0.38	1.20	*
G. candidum	GECA22s00494g	similar to Saccharomyces cerevisiae YLR438W CAR2 L-ornithine transaminase (OTAse), catalyzes the second step of arginine degradation	0.00	1.29	1.83	4.82	*
G. candidum	GECA05s02012g	similar to Saccharomyces cerevisiae YER023W PRO3 Delta 1-pyrroline-5-carboxylate reductase, catalyzes the last step in proline biosynthesis	0.00	0.09	0.09	-1.32	*
D. hansenii	DEHA2G24706g	similar to Saccharomyces cerevisiae YPL111W CAR1 Arginase	0.00	-1.61	-1.24	-2.03	*
D. hansenii	DEHA2F06006g	similar to Saccharomyces cerevisiae YLR438W CAR2 L-ornithine transaminase (OTAse), catalyzes the second step of arginine degradation	0.00	-1.49	-0.75	-0.71	*
D. hansenii	DEHA2F00682g	weakly similar to Saccharomyces cerevisiae YLR438W CAR2 L-ornithine transaminase (OTAse), catalyzes the second step of arginine degradation	0.00	-1.12	-0.14	-0.77	*
D. hansenii	DEHA2F06182g	similar to Saccharomyces cerevisiae YER023W PRO3 Delta 1-pyrroline-5-carboxylate reductase, catalyzes the last step in proline biosynthesis	0.00	0.44	0.29	-0.06	
**Catabolism of proline to glutamate:**							
G. candidum	GECA11s02474g	similar to Saccharomyces cerevisiae YLR142W PUT1 Proline oxidase, nuclear-encoded mitochondrial protein involved in utilization of proline as sole nitrogen source	0.00	1.15	1.52	1.59	*
G. candidum	GECA08s04443g	similar to Saccharomyces cerevisiae YHR037W PUT2, Delta-1-pyrroline-5-carboxylate dehydrogenase	0.00	1.19	1.71	0.13	*
D. hansenii	DEHA2E17952g	similar to CA1552 Candida albicans PUT1 Proline oxidase	0.00	-2.42	-2.89	-0.03	
D. hansenii	DEHA2G20482g	similar to Saccharomyces cerevisiae YHR037W PUT2, Delta-1-pyrroline-5-carboxylate dehydrogenase	0.00	-2.03	-2.05	0.22	
**Catabolism of glycine:**							
G. candidum	GECA14s01583g	similar to Saccharomyces cerevisiae YDR019C GCV1 T subunit of the mitochondrial glycine decarboxylase complex	0.00	0.55	1.05	2.86	*
G. candidum	GECA01s10845g	similar to Saccharomyces cerevisiae YDR019C GCV1 T subunit of the mitochondrial glycine decarboxylase complex	0.00	-0.14	0.09	2.16	*
G. candidum	GECA12s03255g	similar to Saccharomyces cerevisiae YAL044C GCV3 H subunit of the mitochondrial glycine decarboxylase complex	0.00	0.31	0.55	0.40	*
D. hansenii	DEHA2C04708g	similar to Saccharomyces cerevisiae YDR019C GCV1 T subunit of the mitochondrial glycine decarboxylase complex	0.00	-0.90	-0.63	2.15	*
D. hansenii	DEHA2F03806g	similar to Saccharomyces cerevisiae YDR019C GCV1 T subunit of the mitochondrial glycine decarboxylase complex	0.00	-1.90	-1.76	0.88	*
D. hansenii	DEHA2D05368g	similar to Saccharomyces cerevisiae YAL044C GCV3 H subunit of the mitochondrial glycine decarboxylase complex	0.00	-0.68	-0.54	0.26	*
**Catabolism of serine and threonine:**							
G. candidum	GECA15s03233g	similar to Saccharomyces cerevisiae YCL064C CHA1 Catabolic L-serine (L-threonine) deaminase, catalyzes the degradation of both L-serine and L-threonine	0.00	-0.25	3.13	7.23	*
D. hansenii	DEHA2A02090g	similar to Saccharomyces cerevisiae YCL064C CHA1 Catabolic L-serine (L-threonine) deaminase, catalyzes the degradation of both L-serine and L-threonine	0.00	-3.10	-2.60	2.22	*
**Transaminases:**							
G. candidum	GECA04s04949g	similar to Saccharomyces cerevisiae YLR027C AAT2 Cytosolic aspartate aminotransferase, involved in nitrogen metabolism	0.00	0.39	0.85	3.80	*
G. candidum	GECA03s00780g	similar to Saccharomyces cerevisiae YLR027C AAT2 Cytosolic aspartate aminotransferase, involved in nitrogen metabolism	0.00	-0.02	-0.30	-0.71	
G. candidum	GECA03s05400g	similar to Saccharomyces cerevisiae YGL202W ARO8 Aromatic aminotransferase I	0.00	0.95	1.87	2.82	*
G. candidum	GECA27s00967g	similar to Saccharomyces cerevisiae YGL202W ARO8 Aromatic aminotransferase I	0.00	-0.09	-0.67	-0.55	
G. candidum	GECA13s01825g	similar to Saccharomyces cerevisiae YLR089C ALT1 Alanine transaminase (glutamic pyruvic transaminase)	0.00	-0.83	-1.46	-3.04	*
G. candidum	GECA08s02584g	similar to Saccharomyces cerevisiae YHR208W BAT1 Mitochondrial branched-chain amino acid (BCAA) aminotransferase preferentially involved in BCAA biosynthesis	0.00	0.21	-0.19	-1.67	*
G. candidum	GECA01s10493g	similar to Saccharomyces cerevisiae YJR148W BAT2 Cytosolic branched-chain amino acid (BCAA) aminotransferase	0.00	0.43	-0.07	0.33	*
G. candidum	GECA02s09327g	similar to Saccharomyces cerevisiae YFL030W AGX1 Alanine:glyoxylate aminotransferase (AGT)	0.00	-0.04	-0.85	2.11	*
G. candidum	GECA12s04355g	similar to Saccharomyces cerevisiae YFL030W AGX1 Alanine:glyoxylate aminotransferase (AGT)	0.00	0.10	0.07	-0.61	

		Fold change calculated from cumulated reads of G. candidum transaminases:	***0.00***	***0.09***	***-0.11***	***1.18***	
D. hansenii	DEHA2E03630g	similar to Saccharomyces cerevisiae YLR027C AAT2 Cytosolic aspartate aminotransferase, involved in nitrogen metabolism	0.00	0.55	0.59	0.69	*
D. hansenii	DEHA2C05236g	similar to Saccharomyces cerevisiae YLR027C AAT2 Cytosolic aspartate aminotransferase, involved in nitrogen metabolism	0.00	0.44	0.36	0.22	
D. hansenii	DEHA2B03014g	similar to Saccharomyces cerevisiae YLR027C AAT2 Cytosolic aspartate aminotransferase, involved in nitrogen metabolism	0.00	0.35	0.73	3.04	*
D. hansenii	DEHA2A06886g	similar to Saccharomyces cerevisiae YGL202W ARO8 Aromatic aminotransferase I	0.00	0.16	0.57	2.13	*
D. hansenii	DEHA2B08382g	similar to Saccharomyces cerevisiae YLR089C ALT1 Alanine transaminase (glutamic pyruvic transaminase)	0.00	-0.76	-0.15	0.54	*
D. hansenii	DEHA2D06952g	similar to Saccharomyces cerevisiae YJR148W BAT2 Cytosolic branched-chain amino acid (BCAA) aminotransferase	0.00	0.08	-0.03	1.31	*
D. hansenii	DEHA2E01694g	similar to Saccharomyces cerevisiae YFL030W AGX1 Alanine:glyoxylate aminotransferase (AGT)	0.00	0.20	0.08	0.86	*
		Fold change calculated from cumulated reads of D. hansenii transaminases:	***0.00***	***0.34***	***0.32***	***0.86***	
**Amino acid permeases:**							
G. candidum	GECA01s10427g	similar to Saccharomyces cerevisiae YKR039W GAP1 General amino acid permease	0.00	0.74	0.88	2.00	*
G. candidum	GECA03s01374g	similar to Saccharomyces cerevisiae YKR039W GAP1 General amino acid permease	0.00	1.51	2.02	1.75	*
G. candidum	GECA10s01209g	similar to Saccharomyces cerevisiae YKR039W GAP1 General amino acid permease	0.00	-1.97	-4.28	-2.29	*
G. candidum	GECA01s10394g	similar to Saccharomyces cerevisiae YKR039W GAP1 General amino acid permease	0.00	0.71	0.95	0.86	*
G. candidum	GECA19s00989g	similar to Saccharomyces cerevisiae YKR039W GAP1 General amino acid permease	0.00	-1.25	-0.38	-2.08	*
G. candidum	GECA21s00912g	similar to Saccharomyces cerevisiae YEL063C CAN1 Plasma membrane arginine permease	0.00	0.21	0.66	0.14	*
G. candidum	GECA13s03145g	similar to Saccharomyces cerevisiae YEL063C CAN1 Plasma membrane arginine permease	0.00	-0.33	-0.51	-0.68	
G. candidum	GECA16s00153g	similar to Saccharomyces cerevisiae YFL055W AGP3 Low-affinity amino acid permease	0.00	0.97	0.22	-0.25	
G. candidum	GECA04s07644g	similar to Saccharomyces cerevisiae YFL055W AGP3 Low-affinity amino acid permease	0.00	-0.27	-0.26	-0.93	
G. candidum	GECA04s07638g	similar to Saccharomyces cerevisiae YFL055W AGP3 Low-affinity amino acid permease	0.00	-0.46	-1.29	-1.66	*
G. candidum	GECA03s05202g	similar to Saccharomyces cerevisiae YGR055W MUP1 High affinity methionine permease	0.00	-1.23	-1.23	1.97	*
G. candidum	GECA09s02804g	similar to Saccharomyces cerevisiae YGR055W MUP1 High affinity methionine permease	0.00	0.20	-0.17	0.26	*
G. candidum	GECA14s00417g	similar to Saccharomyces cerevisiae YHL036W MUP3 Low affinity methionine permease	0.00	0.37	0.66	-0.15	*
G. candidum	GECA04s03530g	similar to Saccharomyces cerevisiae YOR348C PUT4 Proline permease, required for high-affinity transport of proline	0.00	0.53	0.18	0.49	*
G. candidum	GECA01s08513g	similar to Saccharomyces cerevisiae YPL265W DIP5 Dicarboxylic amino acid permease	0.00	0.40	0.37	-2.33	*
G. candidum	GECA12s03211g	conserved hypothetical protein. Putative amino acid permease	0.00	-0.20	-1.05	-2.50	*
G. candidum	GECA04s04553g	similar to Saccharomyces cerevisiae YPR021C AGC1 Mitochondrial amino acid transporter	0.00	-0.65	-1.52	-3.11	*
		Fold change calculated from cumulated reads of G. candidum amino acid permeases:	***0.00***	***0.20***	***0.20***	***0.13***	
D. hansenii	DEHA2C12056g	similar to Saccharomyces cerevisiae YKR039W GAP1 General amino acid permease	0.00	-3.60	-2.60	-3.52	*
D. hansenii	DEHA2E10076g	similar to Saccharomyces cerevisiae YKR039W GAP1 General amino acid permease	0.00	1.25	1.21	-1.31	*
D. hansenii	DEHA2G06930g	similar to Saccharomyces cerevisiae YKR039W GAP1 General amino acid permease	0.00	-0.63	-0.15	0.91	*
D. hansenii	DEHA2C07194g	similar to Saccharomyces cerevisiae YKR039W GAP1 General amino acid permease	0.00	-0.01	0.06	0.80	*
D. hansenii	DEHA2E11308g	similar to Saccharomyces cerevisiae YKR039W GAP1 General amino acid permease	0.00	0.79	0.63	0.11	
D. hansenii	DEHA2F20944g	similar to Saccharomyces cerevisiae YEL063C CAN1 Plasma membrane arginine permease	0.00	0.07	-0.13	-1.55	*
D. hansenii	DEHA2G11572g	similar to Saccharomyces cerevisiae YFL055W AGP3 Low-affinity amino acid permease	0.00	0.12	0.11	0.27	*
D. hansenii	DEHA2C03102g	similar to Saccharomyces cerevisiae YGR055W MUP1 High affinity methionine permease	0.00	1.21	1.04	-1.02	*
D. hansenii	DEHA2F05566g	similar to Saccharomyces cerevisiae YGR055W MUP1 High affinity methionine permease	0.00	0.10	0.44	0.53	
D. hansenii	DEHA2B01078g	similar to Saccharomyces cerevisiae YOR348C PUT4 Proline permease, required for high-affinity transport of proline	0.00	-1.58	-0.70	2.68	*
D. hansenii	DEHA2C07832g	similar to Saccharomyces cerevisiae YPL265W DIP5 Dicarboxylic amino acid permease	0.00	-0.33	0.18	-0.28	
D. hansenii	DEHA2G07216g	similar to uniprot|P48813 Saccharomyces cerevisiae YDR508C GNP1 High-affinity glutamine permease	0.00	-0.68	-0.53	0.19	
D. hansenii	DEHA2A00748g	similar to uniprot|P48813 Saccharomyces cerevisiae YDR508C GNP1 High-affinity glutamine permease	0.00	-2.47	-1.38	3.01	*
D. hansenii	DEHA2G14608g	similar to uniprot|P32487 Saccharomyces cerevisiae YNL268W LYP1 Lysine permease	0.00	0.58	0.05	2.54	*
D. hansenii	DEHA2G04994g	similar to uniprot|P41815 Saccharomyces cerevisiae YDR046C BAP3 Amino acid permease involved in the uptake of cysteine leucine isoleucine and valine	0.00	0.85	0.87	-0.08	
D. hansenii	DEHA2D11418g	weakly similar to uniprot|P46674 Saccharomyces cerevisiae YDR159W SAC3 Leucine permease transcriptional regulator	0.00	0.55	0.98	0.15	
D. hansenii	DEHA2G07172g	similar to uniprot|Q5KIR6 Cryptococcus neoformans var CND02020 or uniprot|Q5KF59 Cryptococcus neoformans var CNF02890 Putative neutral amino acid transporter	0.00	-2.70	-2.64	-0.13	
D. hansenii	DEHA2A04796g	similar to uniprot|P47040 Saccharomyces cerevisiae YJL059w YHC3 basic amino acid transporter activity	0.00	0.20	-0.12	-1.16	
		Fold change calculated from cumulated reads of D. hansenii amino acid permeases:	***0.00***	***0.03***	***0.11***	***0.07***	
**Secreted lipases and beta-oxidation:**							
G. candidum	GECA03s04685g	putative Carboxylesterase/lipase family protein	0.00	-3.12	-3.55	-3.99	*

G. candidum	GECA03s04718g	Galactomyces geotrichum clone L107 lipase gene, JX074060	0.00	-2.96	-3.81	-3.20	*
G. candidum	GECA19s01352g	similar to Saccharomyces cerevisiae YBR041W FAT1 Very long chain fatty acyl-CoA synthetase and long chain fatty acid transporter	0.00	-0.86	-0.87	-1.39	*
G. candidum	GECA16s03057g	similar to Saccharomyces cerevisiae YER015W FAA2 Medium chain fatty acyl-CoA synthetase	0.00	0.60	0.24	1.49	*
G. candidum	GECA10s03167g	similar to Saccharomyces cerevisiae YOR317W FAA1 Long chain fatty acyl-CoA synthetase	0.00	0.33	0.44	-1.42	*
G. candidum	GECA21s01495g	similar to Saccharomyces cerevisiae YOR317W FAA1 Long chain fatty acyl-CoA synthetase	0.00	0.17	0.26	1.59	*
G. candidum	GECA02s09052g	similar to Saccharomyces cerevisiae YMR246W FAA4 Long chain fatty acyl-CoA synthetase	0.00	0.59	0.85	-0.66	*
G. candidum	GECA10s01055g	putative acyl-CoA dehydrogenase	0.00	0.44	0.34	-0.77	
G. candidum	GECA04s01726g	putative acyl-CoA dehydrogenase	0.00	0.25	-0.19	-2.01	*
G. candidum	GECA01s07996g	putative acyl-CoA dehydrogenase	0.00	0.41	0.45	-2.29	*
G. candidum	GECA14s03310g	similar to Saccharomyces cerevisiae YGL205W POX1 Fatty-acyl coenzyme A oxidase	0.00	0.26	-0.20	-0.34	
G. candidum	GECA27s00395g	similar to Saccharomyces cerevisiae YGL205W POX1 Fatty-acyl coenzyme A oxidase	0.00	0.27	0.38	-1.45	*
G. candidum	GECA10s02848g	similar to Saccharomyces cerevisiae YKR009C FOX2 Multifunctional enzyme of the peroxisomal fatty acid beta-oxidation pathway	0.00	0.81	1.27	1.48	*
G. candidum	GECA07s05048g	similar to Saccharomyces cerevisiae YKR009C FOX2 Multifunctional enzyme of the peroxisomal fatty acid beta-oxidation pathway	0.00	-0.57	-0.69	-2.24	*
G. candidum	GECA16s01759g	similar to Saccharomyces cerevisiae YKR009C FOX2 Multifunctional enzyme of the peroxisomal fatty acid beta-oxidation pathway	0.00	0.27	0.16	-1.46	*
G. candidum	GECA19s00758g	putative enoyl-CoA hydratase	0.00	0.35	0.23	-1.45	*
G. candidum	GECA19s00769g	putative enoyl-CoA hydratase	0.00	0.43	0.26	-1.59	*
G. candidum	GECA11s01451g	similar to Saccharomyces cerevisiae YIL160C POT1 3-ketoacyl-CoA thiolase with broad chain length specificity	0.00	0.48	0.18	0.11	*
G. candidum	GECA07s03772g	similar to Saccharomyces cerevisiae YLR284C ECI1 Peroxisomal delta3,delta2-enoyl-CoA isomerase	0.00	0.46	-0.01	-1.19	*
G. candidum	GECA04s00219g	similar to Saccharomyces cerevisiae YJR019C TES1 Peroxisomal acyl-CoA thioesterase likely to be involved in fatty acid oxidation rather than fatty acid synthesis	0.00	0.68	0.36	1.46	*
G. candidum	GECA20s00967g	similar to Saccharomyces cerevisiae YJR019C TES1 Peroxisomal acyl-CoA thioesterase likely to be involved in fatty acid oxidation rather than fatty acid synthesis	0.00	0.42	0.24	-1.44	*
G. candidum	GECA02s07259g	similar to Saccharomyces cerevisiae YNL202W SPS19 Peroxisomal 2,4-dienoyl-CoA reductase	0.00	0.68	0.72	-1.89	*
G. candidum	GECA05s01022g	similar to Saccharomyces cerevisiae YPR128C ANT1 Peroxisomal adenine nucleotide transporter, involved in beta-oxidation of medium-chain fatty acid	0.00	0.21	0.11	-1.38	*
G. candidum	GECA02s03871g	similar to Saccharomyces cerevisiae YPR128C ANT1 Peroxisomal adenine nucleotide transporter, involved in beta-oxidation of medium-chain fatty acid	0.00	0.54	0.35	-2.60	*
G. candidum	GECA05s07248g	similar to Saccharomyces cerevisiae YOL147C PEX11 Peroxisomal membrane protein required for medium-chain fatty acid oxidation and peroxisome proliferation	0.00	0.54	0.30	-2.06	*
		Fold change calculated from cumulated reads of G. candidum genes involved in fatty acid catabolism:	***0.00***	***0.18***	***0.04***	***-0.70***	
D. hansenii	DEHA2E05940g	weakly similar to uniprot|P32948 Candida rugosa LIP4 Lipase 4 precursor	0.00	0.99	1.36	1.11	*
D. hansenii	DEHA2C01056g	similar to gi|66846736|gb|EAL87068.1| Aspergillus fumigatus Af293 putative lipase	0.00	0.67	1.17	0.57	
D. hansenii	DEHA2D12122g	similar to uniprot|O94091 Candida albicans LIP1 Lipase 1 precursor	0.00	-0.48	-1.07	-1.42	*
D. hansenii	DEHA2A00264g	similar to uniprot|O94091 Candida albicans LIP1 Lipase 1 precursor	0.00	-0.38	-0.22	0.70	
D. hansenii	DEHA2F15224g	similar to Saccharomyces cerevisiae YBR041W FAT1 Very long chain fatty acyl-CoA synthetase and long chain fatty acid transporter	0.00	-0.74	-0.74	-2.68	*
D. hansenii	DEHA2D10076g	similar to Saccharomyces cerevisiae YER015W FAA2 Medium chain fatty acyl-CoA synthetase	0.00	-1.17	-0.93	-2.07	*
D. hansenii	DEHA2B10120g	similar to Saccharomyces cerevisiae YER015W FAA2 Medium chain fatty acyl-CoA synthetase	0.00	-0.35	0.01	-0.99	*
D. hansenii	DEHA2D10098g	similar to Saccharomyces cerevisiae YER015W FAA2 Medium chain fatty acyl-CoA synthetase	0.00	0.65	1.02	0.28	
D. hansenii	DEHA2B11682g	similar to Saccharomyces cerevisiae YER015W FAA2 Medium chain fatty acyl-CoA synthetase	0.00	0.55	0.60	0.02	
D. hansenii	DEHA2G05940g	similar to Saccharomyces cerevisiae YOR317W FAA1 Long chain fatty acyl-CoA synthetase	0.00	-0.19	-0.21	-0.03	
D. hansenii	DEHA2D17248g	similar to Saccharomyces cerevisiae YGL205W POX1 Fatty-acyl coenzyme A oxidase	0.00	-1.01	-0.62	-2.01	*
D. hansenii	DEHA2D17204g	similar to Saccharomyces cerevisiae YGL205W POX1 Fatty-acyl coenzyme A oxidase	0.00	0.26	0.74	0.67	*
D. hansenii	DEHA2C01078g	similar to uniprot|P05335 Candida maltosa Acyl- coenzyme A oxidase POX4 EC 1.3.3.6 Acyl-CoA oxidase	0.00	0.55	1.21	-0.80	*
D. hansenii	DEHA2A08646g	similar to Saccharomyces cerevisiae YKR009C FOX2 Multifunctional enzyme of the peroxisomal fatty acid beta-oxidation pathway	0.00	0.40	0.71	-0.64	*
D. hansenii	DEHA2D18480g	similar to Saccharomyces cerevisiae YIL160C POT1 3-ketoacyl-CoA thiolase with broad chain length specificity	0.00	0.06	0.29	1.18	*
D. hansenii	DEHA2B16368g	similar to Saccharomyces cerevisiae YIL160C POT1 3-ketoacyl-CoA thiolase with broad chain length specificity	0.00	1.27	1.81	2.07	*
D. hansenii	DEHA2E18282g	similar to Saccharomyces cerevisiae YIL160C POT1 3-ketoacyl-CoA thiolase with broad chain length specificity	0.00	-0.15	0.19	-0.96	*
D. hansenii	DEHA2E02024g	similar to Saccharomyces cerevisiae YLR284C ECI1 Peroxisomal delta3,delta2-enoyl-CoA isomerase	0.00	-0.55	-0.21	-1.31	*
D. hansenii	DEHA2A00462g	similar to Saccharomyces cerevisiae YLR284C ECI1 Peroxisomal delta3,delta2-enoyl-CoA isomerase	0.00	1.20	1.69	-1.17	*
D. hansenii	DEHA2E10560g	weakly similar to ca|CA1608|CaTES12 Candida albicans CaTES12 Thiosterase	0.00	0.53	0.86	-2.44	*
D. hansenii	DEHA2A00440g	similar to Saccharomyces cerevisiae YJR019C TES1 Peroxisomal acyl-CoA thioesterase likely to be involved in fatty acid oxidation rather than fatty acid synthesis	0.00	0.84	1.13	-1.53	*
D. hansenii	DEHA2E08910g	similar to Saccharomyces cerevisiae YNL202W SPS19 Peroxisomal 2,4-dienoyl-CoA reductase	0.00	-0.26	-0.65	-0.58	
D. hansenii	DEHA2G22198g	similar to Saccharomyces cerevisiae YNL202W SPS19 Peroxisomal 2,4-dienoyl-CoA reductase	0.00	-0.41	-0.30	-0.90	*
D. hansenii	DEHA2C02178g	similar to Saccharomyces cerevisiae YNL202W SPS19 Peroxisomal 2,4-dienoyl-CoA reductase	0.00	-0.25	0.12	-1.96	*
D. hansenii	DEHA2E08866g	similar to Saccharomyces cerevisiae YNL202W SPS19 Peroxisomal 2,4-dienoyl-CoA reductase	0.00	0.13	0.04	-1.96	*
D. hansenii	DEHA2B10956g	similar to Saccharomyces cerevisiae YNL202W SPS19 Peroxisomal 2,4-dienoyl-CoA reductase	0.00	0.62	1.20	-0.62	

D. hansenii	DEHA2E08316g	similar to Saccharomyces cerevisiae YPR128C ANT1 Peroxisomal adenine nucleotide transporter, involved in beta-oxidation of medium-chain fatty acid	0.00	0.01	0.29	-1.46	*
D. hansenii	DEHA2G13596g	similar to Saccharomyces cerevisiae YPR128C ANT1 Peroxisomal adenine nucleotide transporter, involved in beta-oxidation of medium-chain fatty acid	0.00	0.56	1.00	-0.88	
		Fold change calculated from cumulated reads of D. hansenii genes involved in fatty acid catabolism:	***0.00***	***-0.07***	***0.11***	***-1.33***	
**Catabolism of lactose:**							
D. hansenii	DEHA2G24882g	similar to uniprot|P07921 Kluyveromyces lactis LAC12 Lactose permease	0.00	2.48	2.99	-0.04	
D. hansenii	DEHA2G24860g	similar to uniprot|P00723 Kluyveromyces lactis LAC4 Beta- galactosidase	0.00	0.86	1.16	0.28	
**Catabolism of galactose:**							
G. candidum	GECA10s00615g	similar to Saccharomyces cerevisiae YBR019C GAL10 UDP-glucose-4-epimerase	0.00	-1.04	-1.44	-2.71	*
G. candidum	GECA18s01066g	similar to Saccharomyces cerevisiae YBR019C GAL10 UDP-glucose-4-epimerase	0.00	-0.04	-0.09	0.10	*
G. candidum	GECA24s00791g	similar to Saccharomyces cerevisiae YBR020W GAL1 Galactokinase, phosphorylates alpha-D-galactose to alpha-D-galactose-1-phosphate in the first step of galactose catabolism	0.00	-1.46	-1.74	-0.82	
G. candidum	GECA22s01396g	similar to Saccharomyces cerevisiae YBR018C GAL7 Galactose-1-phosphate uridyl transferase	0.00	-0.52	-0.41	0.58	*
G. candidum	GECA22s01286g	similar to Saccharomyces cerevisiae YMR105C PGM2 Phosphoglucomutase, catalyzes the conversion from glucose-1-phosphate to glucose-6-phosphate	0.00	0.22	-0.05	-1.54	*
		Fold change calculated from cumulated reads of G. candidum genes involved in galactose catabolism:	***0.00***	***-0.66***	***-0.83***	***-0.72***	
D. hansenii	DEHA2C02398g	weakly similar to Saccharomyces cerevisiae YBR019C GAL10 UDP-glucose-4-epimerase	0.00	-1.10	-1.55	0.28	
D. hansenii	DEHA2C02464g	similar to Saccharomyces cerevisiae YBR019C GAL10 UDP-glucose-4-epimerase	0.00	-1.48	-2.02	-0.98	*
D. hansenii	DEHA2C02376g	similar to Saccharomyces cerevisiae YBR018C GAL7 Galactose-1-phosphate uridyl transferase	0.00	-1.46	-1.73	-0.37	
D. hansenii	DEHA2C05258g	similar to Saccharomyces cerevisiae YMR105C PGM2 Phosphoglucomutase, catalyzes the conversion from glucose-1-phosphate to glucose-6-phosphate	0.00	0.09	0.20	-0.45	*
		Fold change calculated from cumulated reads of D. hansenii genes involved in galactose catabolism:	***0.00***	***-1.03***	***-1.25***	***-0.69***	
Catabolism of lactate:							
G. candidum	GECA32s02793g	similar to Saccharomyces cerevisiae YKL217W JEN1 Monocarboxylate/proton symporter of the plasma membrane	0.00	0.32	-0.18	2.80	*
G. candidum	GECA23s00043g	similar to Saccharomyces cerevisiae YKL217W JEN1 Monocarboxylate/proton symporter of the plasma membrane	0.00	-3.06	2.66	7.73	*
G. candidum	GECA20s00164g	similar to Saccharomyces cerevisiae YNL125C ESBP6 Protein with similarity to monocarboxylate permeases	0.00	-1.28	-0.17	-0.34	
G. candidum	GECA06s04828g	similar to Saccharomyces cerevisiae YDL174C DLD1 D-lactate dehydrogenase, oxidizes D-lactate to pyruvate	0.00	-0.25	-0.37	0.40	*
G. candidum	GECA06s02133g	similar to Saccharomyces cerevisiae YDL174C DLD1 D-lactate dehydrogenase, oxidizes D-lactate to pyruvate	0.00	-0.09	0.07	-1.09	*
G. candidum	GECA06s00549g	similar to Saccharomyces cerevisiae YDL178W DLD2 D-lactate dehydrogenase, located in the mitochondrial matrix	0.00	-0.18	-0.27	-0.37	
G. candidum	GECA11s03728g	similar to Saccharomyces cerevisiae YML054C CYB2 Cytochrome b2 (L-lactate cytochrome-c oxidoreductase)	0.00	0.39	0.65	-0.30	
G. candidum	GECA04s07677g	similar to Saccharomyces cerevisiae YML054C CYB2 Cytochrome b2 (L-lactate cytochrome-c oxidoreductase)	0.00	0.57	0.39	-0.49	
G. candidum	GECA18s01000g	similar to Saccharomyces cerevisiae YML054C CYB2 Cytochrome b2 (L-lactate cytochrome-c oxidoreductase)	0.00	0.33	-0.06	-1.69	*
		Fold change calculated from cumulated reads of G. candidum genes involved in lactate catabolism:	***0.00***	***0.11***	***0.16***	***2.14***	
D. hansenii	DEHA2F17402g	similar to Saccharomyces cerevisiae YKL217W JEN1 Monocarboxylate/proton symporter of the plasma membrane	0.00	2.33	2.89	2.06	*
D. hansenii	DEHA2E24024g	similar to Saccharomyces cerevisiae YKL217W JEN1 Monocarboxylate/proton symporter of the plasma membrane	0.00	1.10	0.72	1.15	*
D. hansenii	DEHA2D18920g	similar to Saccharomyces cerevisiae YKL217W JEN1 Monocarboxylate/proton symporter of the plasma membrane	0.00	-1.68	-2.27	-1.73	*
D. hansenii	DEHA2E09944g	similar to Saccharomyces cerevisiae YNL125C ESBP6 Protein with similarity to monocarboxylate permeases	0.00	0.91	1.66	2.91	*
D. hansenii	DEHA2D08734g	similar to Saccharomyces cerevisiae YDL174C DLD1 D-lactate dehydrogenase, oxidizes D-lactate to pyruvate	0.00	0.55	0.32	0.37	
D. hansenii	DEHA2F06930g	similar to Saccharomyces cerevisiae YDL174C DLD1 D-lactate dehydrogenase, oxidizes D-lactate to pyruvate	0.00	0.49	0.60	-0.21	
D. hansenii	DEHA2B10142g	similar to Saccharomyces cerevisiae YDL178W DLD2 D-lactate dehydrogenase, located in the mitochondrial matrix	0.00	0.06	0.17	-0.05	
D. hansenii	DEHA2E00836g	similar to Saccharomyces cerevisiae YML054C CYB2 Cytochrome b2 (L-lactate cytochrome-c oxidoreductase)	0.00	0.91	0.83	-0.29	
D. hansenii	DEHA2D05522g	similar to Saccharomyces cerevisiae YML054C CYB2 Cytochrome b2 (L-lactate cytochrome-c oxidoreductase)	0.00	-0.32	-0.07	-0.76	*
		Fold change calculated from cumulated reads of D. hansenii genes involved in lactate catabolism:	***0.00***	***1.20***	***1.33***	***1.10***	
**NADH:ubiquinone oxidoreductase (complex I):**							
G. candidum	GECA_mCDS2187	NADH dehydrogenase subunit 1	0.00	0.08	0.17	-1.58	*
G. candidum	GECA_mCDS1810	NADH dehydrogenase subunit 2	0.00	0.48	0.76	-0.79	
G. candidum	GECA_mCDS1976	NADH dehydrogenase subunit 3	0.00	0.50	0.75	-0.83	
G. candidum	GECA_mCDS4506	NADH dehydrogenase subunit 4	0.00	0.31	0.37	-1.10	*
G. candidum	GECA_mCDS9794	NADH dehydrogenase subunit 5	0.00	0.30	0.34	-0.37	
G. candidum	GECA_mCDS2083	NADH dehydrogenase subunit 6	0.00	0.11	0.19	-1.68	*

G. candidum	GECA02s04916g	N7BM subunit of mitochondrial NADH:ubiquinone oxidoreductase (complex I), putative	0.00	-0.37	-0.80	-3.19	*
G. candidum	GECA11s01638g	NB2M subunit of mitochondrial NADH:ubiquinone oxidoreductase (complex I), putative	0.00	-0.43	-0.49	-1.27	*
G. candidum	GECA13s02562g	NB4M subunit of mitochondrial NADH:ubiquinone oxidoreductase (complex I), putative	0.00	-0.50	-0.92	-2.65	*
G. candidum	GECA07s00719g	NB5M subunit of mitochondrial NADH:ubiquinone oxidoreductase (complex I), putative	0.00	-0.38	-0.46	-2.32	*
G. candidum	GECA01s02859g	NB6M subunit of mitochondrial NADH:ubiquinone oxidoreductase (complex I), putative	0.00	-0.61	-0.95	-3.16	*
G. candidum	GECA11s01715g	NB8M subunit of mitochondrial NADH:ubiquinone oxidoreductase (complex I), putative	0.00	-0.44	-0.87	-3.32	*
G. candidum	GECA04s01517g	NESM subunit of mitochondrial NADH:ubiquinone oxidoreductase (complex I), putative	0.00	-0.65	-1.02	-2.30	*
G. candidum	GECA09s00604g	NI2M subunit of mitochondrial NADH:ubiquinone oxidoreductase (complex I), putative	0.00	-0.33	-0.60	-2.65	*
G. candidum	GECA19s00890g	NI8M subunit of mitochondrial NADH:ubiquinone oxidoreductase (complex I), putative	0.00	0.08	-0.09	-1.23	*
G. candidum	GECA02s07908g	NI8M subunit of mitochondrial NADH:ubiquinone oxidoreductase (complex I), putative	0.00	-0.45	-0.62	-2.36	*
G. candidum	GECA06s05081g	NI9M subunit of mitochondrial NADH:ubiquinone oxidoreductase (complex I), putative	0.00	-0.40	-0.88	-2.39	*
G. candidum	GECA03s00571g	NIAM subunit of mitochondrial NADH:ubiquinone oxidoreductase (complex I), putative	0.00	-0.47	-0.68	-1.64	*
G. candidum	GECA05s00164g	NIDM subunit of mitochondrial NADH:ubiquinone oxidoreductase (complex I), putative	0.00	-0.49	-0.86	-3.05	*
G. candidum	GECA08s03189g	NIMM subunit of mitochondrial NADH:ubiquinone oxidoreductase (complex I), putative	0.00	-0.73	-1.16	-2.31	*
G. candidum	GECA20s01044g	NIPM subunit of mitochondrial NADH:ubiquinone oxidoreductase (complex I), putative	0.00	-0.51	-0.80	-3.03	*
G. candidum	GECA17s02276g	NUAM subunit of mitochondrial NADH:ubiquinone oxidoreductase (complex I), putative	0.00	-0.75	-1.27	-1.83	*
G. candidum	GECA08s04113g	NUAM subunit of mitochondrial NADH:ubiquinone oxidoreductase (complex I), putative	0.00	-0.39	-1.06	-3.18	*
G. candidum	GECA04s04729g	NUBM subunit of mitochondrial NADH:ubiquinone oxidoreductase (complex I), putative	0.00	-0.70	-1.29	-0.72	
G. candidum	GECA09s02083g	NUCM subunit of mitochondrial NADH:ubiquinone oxidoreductase (complex I), putative	0.00	-0.57	-0.92	-0.95	*
G. candidum	GECA12s01440g	NUEM subunit of mitochondrial NADH:ubiquinone oxidoreductase (complex I), putative	0.00	-0.40	-0.57	-1.27	*
G. candidum	GECA03s07314g	NUFM subunit of mitochondrial NADH:ubiquinone oxidoreductase (complex I), putative	0.00	-0.44	-0.73	-2.04	*
G. candidum	GECA02s07666g	NUGM subunit of mitochondrial NADH:ubiquinone oxidoreductase (complex I), putative	0.00	-0.68	-1.09	-2.18	*
G. candidum	GECA02s06929g	NUHM subunit of mitochondrial NADH:ubiquinone oxidoreductase (complex I), putative	0.00	-0.52	-0.77	-0.96	*
G. candidum	GECA10s03684g	NUIM subunit of mitochondrial NADH:ubiquinone oxidoreductase (complex I), putative	0.00	-0.48	-0.48	-0.12	*
G. candidum	GECA01s05532g	NUJM subunit of mitochondrial NADH:ubiquinone oxidoreductase (complex I), putative	0.00	-0.65	-0.87	-1.52	*
G. candidum	GECA01s00098g	NUKM subunit of mitochondrial NADH:ubiquinone oxidoreductase (complex I), putative	0.00	-0.25	-0.21	0.39	*
G. candidum	GECA04s05059g	NUMM subunit of mitochondrial NADH:ubiquinone oxidoreductase (complex I), putative	0.00	-0.41	-0.75	-2.68	*
G. candidum	GECA05s05884g	NUPM subunit of mitochondrial NADH:ubiquinone oxidoreductase (complex I), putative	0.00	-0.44	-0.89	-2.85	*
G. candidum	GECA10s02782g	NUXM subunit of mitochondrial NADH:ubiquinone oxidoreductase (complex I), putative	0.00	-0.67	-0.94	-2.64	*
G. candidum	GECA07s00098g	NUYM subunit of mitochondrial NADH:ubiquinone oxidoreductase (complex I), putative	0.00	-0.44	-0.91	-1.95	*
G. candidum	GECA18s02386g	NUZM subunit of mitochondrial NADH:ubiquinone oxidoreductase (complex I), putative	0.00	-0.39	-0.91	-2.67	*
		Fold change calculated from cumulated reads of G. candidum NADH:ubiquinone oxidoreductase subunits:	***0.00***	***-0.10***	***-0.15***	***-1.19***	
D. hansenii	DEHA2A_mCDS39	NADH dehydrogenase subunit 1	0.00	-0.13	-0.29	-1.11	*
D. hansenii	DEHA2A_mCDS50	NADH dehydrogenase subunit 2	0.00	-0.99	-1.49	-2.82	*
D. hansenii	DEHA2A_mCDS63	NADH dehydrogenase subunit 3	0.00	-1.02	-1.52	-3.02	*
D. hansenii	DEHA2A_mCDS26	NADH dehydrogenase subunit 4	0.00	-0.48	-0.57	-2.30	*
D. hansenii	DEHA2A_mCDS16	NADH dehydrogenase subunit 4L	0.00	0.20	-0.03	-0.58	*
D. hansenii	DEHA2A_mCDS14	NADH dehydrogenase subunit 5	0.00	0.42	-0.03	-0.76	*
D. hansenii	DEHA2A_mCDS34	NADH dehydrogenase subunit 6	0.00	-0.15	-0.37	-1.23	*
D. hansenii	DEHA2D02948g	highly similar to uniprot|Q9UUT8 Yarrowia lipolytica YALI0F00924g nuim Subunit NUIM of protein NADH:Ubiquinone Oxidoreductase	0.00	-1.55	-1.42	-0.37	
D. hansenii	DEHA2F02552g	highly similar to uniprot|Q9UUT7 Yarrowia lipolytica nukm Subunit NUKM of protein NADH:Ubiquinone Oxidoreductase precursor	0.00	-1.68	-1.46	-0.57	*
D. hansenii	DEHA2A06820g	highly similar to uniprot|Q9UUT9 Yarrowia lipolytica YALI0D00737g nuhm Subunit NUHM of protein NADH:Ubiquinone Oxidoreductase (Complex I)	0.00	-1.32	-1.20	-0.67	*
		Fold change calculated from cumulated reads of D. hansenii NADH:ubiquinone oxidoreductase subunits:	***0.00***	***-0.17***	***-0.48***	***-1.27***	
**Succinate dehydrogenase (ubiquinone) (complex II) :**							
G. candidum	GECA04s02518g	similar to Saccharomyces cerevisiae YKL148C SDH1 Flavoprotein subunit of succinate dehydrogenase	0.00	-0.38	-0.58	-0.57	
G. candidum	GECA02s04223g	similar to Saccharomyces cerevisiae YLL041C SDH2 Iron-sulfur protein subunit of succinate dehydrogenase (Sdh1p, Sdh2p, Sdh3p, Sdh4p)	0.00	-0.20	-0.32	-0.89	*
G. candidum	GECA17s01968g	similar to Saccharomyces cerevisiae YLL041C SDH2 Iron-sulfur protein subunit of succinate dehydrogenase (Sdh1p, Sdh2p, Sdh3p, Sdh4p)	0.00	-1.74	-2.10	-1.03	*
G. candidum	GECA03s03717g	similar to Saccharomyces cerevisiae YKL141W SDH3 Subunit of succinate dehydrogenase and of TIM22 translocase	0.00	-0.17	-0.46	-0.39	
G. candidum	GECA19s01473g	similar to Saccharomyces cerevisiae YDR178W SDH4 Membrane anchor subunit of succinate dehydrogenase (Sdh1p, Sdh2p, Sdh3p, Sdh4p)	0.00	0.17	0.16	-1.37	*
		Fold change calculated from cumulated reads of G. candidum succinate dehydrogenase subunits:	***0.00***	***-0.29***	***-0.46***	***-0.72***	

D. hansenii	DEHA2E07458g	similar to Saccharomyces cerevisiae YKL148C SDH1 Flavoprotein subunit of succinate dehydrogenase	0.00	-0.58	-0.66	0.09	
D. hansenii	DEHA2E08888g	similar to Saccharomyces cerevisiae YKL148C SDH1 Flavoprotein subunit of succinate dehydrogenase	0.00	-0.24	-0.24	0.61	*
D. hansenii	DEHA2G19382g	similar to Saccharomyces cerevisiae YLL041C SDH2 Iron-sulfur protein subunit of succinate dehydrogenase (Sdh1p, Sdh2p, Sdh3p, Sdh4p)	0.00	-2.25	-1.99	0.33	*
D. hansenii	DEHA2G15928g	similar to Saccharomyces cerevisiae YDR178W SDH4 Membrane anchor subunit of succinate dehydrogenase (Sdh1p, Sdh2p, Sdh3p, Sdh4p)	0.00	-1.21	-1.48	0.01	
		Fold change calculated from cumulated reads of D. hansenii succinate dehydrogenase subunits:	***0.00***	***-0.72***	***-0.73***	***0.36***	

Ubiquinol cytochrome c oxidoreductase (complex III)

G. candidum	GECA12s03937g	similar to Saccharomyces cerevisiae YEL024W RIP1 Ubiquinol-cytochrome-c reductase	0.00	-0.50	-0.80	-0.41	
G. candidum	GECA16s00736g	similar to Saccharomyces cerevisiae YOR065W CYT1 Cytochrome c1	0.00	-0.86	-1.44	-1.41	*
G. candidum	GECA17s01610g	similar to Saccharomyces cerevisiae YOR065W CYT1 Cytochrome c1	0.00	-0.31	-0.56	-3.28	*
G. candidum	GECA22s00945g	similar to Saccharomyces cerevisiae YPR191W QCR2 Subunit 2 of the ubiquinol cytochrome-c reductase complex	0.00	-0.28	-0.64	-1.92	*
G. candidum	GECA05s06412g	similar to Saccharomyces cerevisiae YPR191W QCR2 Subunit 2 of the ubiquinol cytochrome-c reductase complex	0.00	-0.60	-1.20	-2.56	*
G. candidum	GECA04s02375g	similar to Saccharomyces cerevisiae YFR033C QCR6 Subunit 6 of the ubiquinol cytochrome-c reductase complex	0.00	-0.39	-0.71	-3.23	*
G. candidum	GECA23s01077g	similar to Saccharomyces cerevisiae YDR529C QCR7 Subunit 7 of the ubiquinol cytochrome-c reductase complex	0.00	-0.46	-0.99	-2.31	*
G. candidum	GECA07s04707g	similar to Saccharomyces cerevisiae YJL166W QCR8 Subunit 8 of ubiquinol cytochrome-c reductase complex	0.00	-0.73	-0.99	-1.73	*
G. candidum	GECA22s01539g	similar to Saccharomyces cerevisiae YGR183C QCR9 Subunit 9 of the ubiquinol cytochrome-c reductase complex	0.00	-0.48	-0.91	-3.47	*
G. candidum	GECA02s05691g	similar to Saccharomyces cerevisiae YHR001W-A QCR10 Subunit of the ubiquinol-cytochrome c oxidoreductase complex	0.00	0.01	-0.49	-1.35	*
		Fold change calculated from cumulated reads of G. candidum ubiquinol cytochrome c oxidoreductase subunits:	***0.00***	***-0.55***	***-0.95***	***-1.66***	
D. hansenii	DEHA2F11726g	similar to Saccharomyces cerevisiae YEL024W RIP1 Ubiquinol-cytochrome-c reductase	0.00	-0.76	-0.85	-1.06	*
D. hansenii	DEHA2C06402g	similar to Saccharomyces cerevisiae YOR065W CYT1 Cytochrome c1	0.00	-1.36	-1.46	-0.88	*
D. hansenii	DEHA2D13640g	similar to uniprot|P07256 Saccharomyces cerevisiae YBL045C COR1 Core subunit of the ubiquinol-cytochrome c reductase complex	0.00	-0.79	-0.90	-1.29	*
D. hansenii	DEHA2E09834g	similar to Saccharomyces cerevisiae YPR191W QCR2 Subunit 2 of the ubiquinol cytochrome-c reductase complex	0.00	-0.99	-1.15	-0.62	*
D. hansenii	DEHA2D11990g	similar to Saccharomyces cerevisiae YFR033C QCR6 Subunit 6 of the ubiquinol cytochrome-c reductase complex	0.00	-0.84	-0.84	-0.93	*
D. hansenii	DEHA2E19756g	similar to Saccharomyces cerevisiae YDR529C QCR7 Subunit 7 of the ubiquinol cytochrome-c reductase complex	0.00	-0.81	-0.94	-0.41	*
D. hansenii	DEHA2F08250g	similar to Saccharomyces cerevisiae YJL166W QCR8 Subunit 8 of ubiquinol cytochrome-c reductase complex	0.00	-0.59	-0.60	0.06	
D. hansenii	DEHA2G18524g	similar to Saccharomyces cerevisiae YGR183C QCR9 Subunit 9 of the ubiquinol cytochrome-c reductase complex	0.00	-0.38	-0.62	0.40	
D. hansenii	DEHA2C08448g	similar to Saccharomyces cerevisiae YHR001W-A QCR10 Subunit of the ubiquinol-cytochrome c oxidoreductase complex	0.00	-1.23	-1.14	-0.59	*
		Fold change calculated from cumulated reads of D. hansenii ubiquinol cytochrome c oxidoreductase subunits:	***0.00***	***-0.88***	***-0.97***	***-0.82***	

Cytochrome c oxidase (complex IV):

G. candidum	GECA_mCDS2416(	cytochrome c oxidase subunit 1	0.00	-0.06	0.16	-1.07	*
G. candidum	GECA_mCDS3524	cytochrome c oxidase subunit 3	0.00	0.14	0.40	-0.56	
G. candidum	GECA08s04146g	similar to Saccharomyces cerevisiae YGL187C COX4 Subunit IV of cytochrome c oxidase	0.00	-0.61	-1.20	-3.05	*
G. candidum	GECA20s01330g	similar to Saccharomyces cerevisiae YNL052W COX5A Subunit Va of cytochrome c oxidase	0.00	-0.67	-1.30	-3.26	*
G. candidum	GECA03s05015g	similar to Saccharomyces cerevisiae YHR051W COX6 Subunit VI of cytochrome c oxidase	0.00	-0.49	-0.98	-1.79	*
G. candidum	GECA13s00637g	similar to Saccharomyces cerevisiae YMR256C COX7 Subunit VII of cytochrome c oxidase	0.00	-0.47	-0.93	-2.24	*
G. candidum	GECA18s01924g	similar to Saccharomyces cerevisiae YLR395C COX8 Subunit VIII of cytochrome c oxidase	0.00	-0.26	-0.66	-2.68	*
G. candidum	GECA18s01913g	similar to Saccharomyces cerevisiae YDL067C COX9 Subunit VIIa of cytochrome c oxidase	0.00	-0.48	-0.87	-2.13	*
G. candidum	GECA12s02826g	similar to Saccharomyces cerevisiae YLR038C COX12 Subunit VIb of cytochrome c oxidase	0.00	-0.42	-0.71	-2.17	*
G. candidum	GECA02s08282g	similar to Saccharomyces cerevisiae YLR038C COX12 Subunit VIb of cytochrome c oxidase	0.00	-0.33	-0.26	-2.30	*
G. candidum	GECA04s00604g	similar to Saccharomyces cerevisiae YGL191W COX13 Subunit VIa of cytochrome c oxidase	0.00	-0.34	-0.70	-2.87	*
		Fold change calculated from cumulated reads of G. candidum cytochrome c oxidase subunits:	***0.00***	***-0.07***	***0.11***	***-1.06***	
D. hansenii	DEHA2A_mCDS	cytochrome c oxidase subunit 1	0.00	-0.22	-0.21	-0.63	*
D. hansenii	DEHA2A_mCDS26	cytochrome c oxidase subunit 2	0.00	-0.08	-0.40	-0.74	
D. hansenii	DEHA2A_mCDS13	cytochrome c oxidase subunit 3	0.00	-0.74	-1.08	-2.01	*
D. hansenii	DEHA2B04664g	similar to Saccharomyces cerevisiae YGL187C COX4 Subunit IV of cytochrome c oxidase	0.00	-1.32	-1.61	-0.91	*
D. hansenii	DEHA2D03036g	similar to Saccharomyces cerevisiae YNL052W COX5A Subunit Va of cytochrome c oxidase	0.00	-0.65	-0.74	-1.07	*
D. hansenii	DEHA2E20658g	similar to Saccharomyces cerevisiae YHR051W COX6 Subunit VI of cytochrome c oxidase	0.00	-0.67	-0.90	-0.82	*
D. hansenii	DEHA2F16302g	similar to Saccharomyces cerevisiae YMR256C COX7 Subunit VII of cytochrome c oxidase	0.00	-0.62	-0.85	-1.14	*

D. hansenii	DEHA2E10648g	weakly similar to Saccharomyces cerevisiae YLR395C COX8 Subunit VIII of cytochrome c oxidase	0.00	-0.92	-1.04	-1.21	*
D. hansenii	DEHA2E10626g	similar to Saccharomyces cerevisiae YDL067C COX9 Subunit VIIa of cytochrome c oxidase	0.00	-0.71	-0.98	-0.90	*
D. hansenii	DEHA2E01628g	similar to Saccharomyces cerevisiae YLR038C COX12 Subunit VIb of cytochrome c oxidase	0.00	-0.94	-1.11	-1.64	*
D. hansenii	DEHA2B04730g	similar to Saccharomyces cerevisiae YGL191W COX13 Subunit VIa of cytochrome c oxidase	0.00	-0.76	-1.28	-0.38	
		Fold change calculated from cumulated reads of D. hansenii cytochrome c oxidase subunits:	***0.00***	***-0.23***	***-0.41***	***-0.75***	
**F1F0 ATP synthase:**							
G. candidum	GECA01s09107g	similar to Saccharomyces cerevisiae YBL099W ATP1 Alpha subunit of the F1 sector of mitochondrial F1F0 ATP synthase	0.00	0.10	-0.22	-2.67	*
G. candidum	GECA04s00593g	similar to Saccharomyces cerevisiae YBL099W ATP1 Alpha subunit of the F1 sector of mitochondrial F1F0 ATP synthase	0.00	0.10	-0.29	-1.56	*
G. candidum	GECA12s03431g	similar to Saccharomyces cerevisiae YJR121W ATP2 Beta subunit of the F1 sector of mitochondrial F1F0 ATP synthase	0.00	-0.06	-0.38	-1.61	*
G. candidum	GECA02s03475g	similar to Saccharomyces cerevisiae YJR121W ATP2 Beta subunit of the F1 sector of mitochondrial F1F0 ATP synthase	0.00	-0.12	-0.51	-2.41	*
G. candidum	GECA14s01572g	similar to Saccharomyces cerevisiae YBR039W ATP3 Gamma subunit of the F1 sector of mitochondrial F1F0 ATP synthase	0.00	0.04	-0.23	-2.29	*
G. candidum	GECA13s01913g	similar to Saccharomyces cerevisiae YBR039W ATP3 Gamma subunit of the F1 sector of mitochondrial F1F0 ATP synthase	0.00	0.08	-0.19	-2.30	*
G. candidum	GECA05s02595g	similar to Saccharomyces cerevisiae YPL078C, ATP4 Subunit b of the stator stalk of mitochondrial F1F0 ATP synthase	0.00	0.00	-0.15	-2.79	*
G. candidum	GECA05s01781g	similar to Saccharomyces cerevisiae YPL078C ATP4, Subunit b of the stator stalk of mitochondrial F1F0 ATP synthase	0.00	0.01	-0.25	-2.89	*
G. candidum	GECA23s01220g	similar to Saccharomyces cerevisiae YDR298C ATP5 Subunit 5 of the stator stalk of mitochondrial F1F0 ATP synthase	0.00	0.16	-0.26	-1.81	*
G. candidum	GECA_mCDS2649	ATPase subunit 6	0.00	0.01	0.20	-0.74	
G. candidum	GECA13s02463g	similar to Saccharomyces cerevisiae YKL016C ATP7 Subunit d of the stator stalk of mitochondrial F1F0 ATP synthase	0.00	0.12	-0.12	-2.97	*
G. candidum	GECA_mCDS2621	ATPase subunit 8	0.00	0.02	0.29	-0.79	
G. candidum	GECA_mCDS7103	ATPase subunit 9	0.00	-0.26	-0.22	-3.21	*
G. candidum	GECA15s00637g	similar to Saccharomyces cerevisiae YLR393W ATP10 Mitochondrial inner membrane protein required for assembly of the F0 sector of mitochondrial F1F0 ATP synthase	0.00	0.11	0.01	-1.57	*
G. candidum	GECA08s00780g	similar to Saccharomyces cerevisiae YLR393W ATP10 Mitochondrial inner membrane protein required for assembly of the F0 sector of mitochondrial F1F0 ATP synthase	0.00	0.04	-0.20	-0.01	*
G. candidum	GECA07s04729g	similar to Saccharomyces cerevisiae YJL180C ATP12 Conserved protein required for assembly of alpha and beta subunits into the F1 sector of mitochondrial F1F0 ATP synthase	0.00	0.06	-0.08	-0.18	*
G. candidum	GECA01s00450g	similar to Saccharomyces cerevisiae YLR295C ATP14 Subunit h of the F0 sector of mitochondrial F1F0 ATP synthase	0.00	0.39	0.82	-1.04	*
G. candidum	GECA15s02155g	similar to Saccharomyces cerevisiae YPL271W ATP15 Epsilon subunit of the F1 sector of mitochondrial F1F0 ATP synthase	0.00	0.03	-0.23	-3.46	*
G. candidum	GECA24s00725g	similar to Saccharomyces cerevisiae YDL004W ATP16 Delta subunit of the central stalk of mitochondrial F1F0 ATP synthase	0.00	0.12	-0.16	-2.35	*
G. candidum	GECA05s00967g	similar to Saccharomyces cerevisiae YDR377W ATP17 Subunit f of the F0 sector of mitochondrial F1F0 ATP synthase	0.00	0.49	0.72	-0.18	
G. candidum	GECA05s07446g	similar to Saccharomyces cerevisiae YML081C-A ATP18 Subunit of the mitochondrial F1F0 ATP synthase	0.00	0.08	-0.09	-2.35	*
G. candidum	GECA01s11615g	similar to Saccharomyces cerevisiae YOL077W-A ATP19 Subunit k of the mitochondrial F1F0 ATP synthase	0.00	0.08	-0.18	-2.61	*
G. candidum	GECA01s05356g	similar to Saccharomyces cerevisiae YPR020W ATP20 Subunit g of the mitochondrial F1F0 ATP synthase	0.00	-0.03	-0.46	-3.32	*
G. candidum	GECA02s03332g	similar to Saccharomyces cerevisiae YDR322C-A TIM11 Subunit of mitochondrial F1F0-ATPase	0.00	0.18	0.18	-1.57	*
		Fold change calculated from cumulated reads of G. candidum F1F0 ATP synthase subunits:	***0.00***	***-0.01***	***-0.07***	***-1.75***	
D. hansenii	DEHA2D13398g	similar to Saccharomyces cerevisiae YBL099W ATP1 Alpha subunit of the F1 sector of mitochondrial F1F0 ATP synthase	0.00	0.34	0.10	-0.34	
D. hansenii	DEHA2F06226g	similar to Saccharomyces cerevisiae YJR121W ATP2 Beta subunit of the F1 sector of mitochondrial F1F0 ATP synthase	0.00	0.47	0.28	-0.31	
D. hansenii	DEHA2F20658g	similar to Saccharomyces cerevisiae YBR039W ATP3 Gamma subunit of the F1 sector of mitochondrial F1F0 ATP synthase	0.00	0.55	0.36	-0.18	
D. hansenii	DEHA2F07942g	similar to Saccharomyces cerevisiae YPL078C, ATP4 Subunit b of the stator stalk of mitochondrial F1F0 ATP synthase	0.00	0.60	0.48	0.44	*
D. hansenii	DEHA2E05500g	similar to Saccharomyces cerevisiae YDR298C ATP5 Subunit 5 of the stator stalk of mitochondrial F1F0 ATP synthase	0.00	0.27	0.19	-0.28	
D. hansenii	DEHA2A_mCDS17	ATPase subunit 6	0.00	0.25	0.17	-0.25	
D. hansenii	DEHA2F25564g	similar to Saccharomyces cerevisiae YKL016C ATP7 Subunit d of the stator stalk of mitochondrial F1F0 ATP synthase	0.00	0.24	0.31	-0.82	*
D. hansenii	DEHA2A_mCDS18	ATPase subunit 8	0.00	0.31	0.16	-0.26	
D. hansenii	DEHA2A_mCDS19	ATPase subunit 9	0.00	0.20	-0.11	-0.52	
D. hansenii	DEHA2A11132g	similar to Saccharomyces cerevisiae YLR393W ATP10 Mitochondrial inner membrane protein required for assembly of the F0 sector of mitochondrial F1F0 ATP synthase	0.00	0.31	0.00	0.39	
D. hansenii	DEHA2F15796g	similar to Saccharomyces cerevisiae YLR393W ATP10 Mitochondrial inner membrane protein required for assembly of the F0 sector of mitochondrial F1F0 ATP synthase	0.00	-0.51	-0.26	0.17	
D. hansenii	DEHA2F02024g	weakly similar to Saccharomyces cerevisiae YLR295C ATP14 Subunit h of the F0 sector of mitochondrial F1F0 ATP synthase	0.00	0.86	0.89	1.18	*
D. hansenii	DEHA2A14014g	similar to Saccharomyces cerevisiae YDL004W ATP16 Delta subunit of the central stalk of mitochondrial F1F0 ATP synthase	0.00	0.81	0.72	0.86	*
D. hansenii	DEHA2D18304g	similar to Saccharomyces cerevisiae YDR377W ATP17 Subunit f of the F0 sector of mitochondrial F1F0 ATP synthase	0.00	0.26	0.38	-0.22	
D. hansenii	DEHA2B07568g	similar to Saccharomyces cerevisiae YML081C-A ATP18 Subunit of the mitochondrial F1F0 ATP synthase	0.00	0.33	0.46	0.19	
D. hansenii	DEHA2G05588g	weakly similar to Saccharomyces cerevisiae YOL077W-A ATP19 Subunit k of the mitochondrial F1F0 ATP synthase	0.00	0.80	0.56	0.97	*
D. hansenii	DEHA2G09680g	similar to Saccharomyces cerevisiae YOL077W-A ATP19 Subunit k of the mitochondrial F1F0 ATP synthase	0.00	-0.01	-0.51	-0.89	*
D. hansenii	DEHA2E16654g	similar to Saccharomyces cerevisiae YPR020W ATP20 Subunit g of the mitochondrial F1F0 ATP synthase	0.00	-0.02	-0.05	-0.75	*
D. hansenii	DEHA2E20504g	similar to Saccharomyces cerevisiae YDR322C-A TIM11 Subunit of mitochondrial F1F0-ATPase	0.00	0.52	0.30	0.27	

		Fold change calculated from cumulated reads of D. hansenii F1F0 ATP synthase subunits:	***0.00***	***0.35***	***0.18***	***-0.26***	
**Iron transport:**							
G. candidum	GECA05s03805g	similar to Saccharomyces cerevisiae YMR058W FET3 Ferro-O2-oxidoreductase required for high-affinity iron uptake and involved in mediating resistance to copper ion toxicity	0.00	0.52	-0.52	-1.61	*
G. candidum	GECA09s00571g	similar to Saccharomyces cerevisiae YMR058W FET3 Ferro-O2-oxidoreductase required for high-affinity iron uptake and involved in mediating resistance to copper ion toxicity	0.00	0.76	1.43	-1.14	*
G. candidum	GECA01s05268g	similar to Saccharomyces cerevisiae YKL220C FRE2 Ferric reductase and cupric reductase	0.00	0.47	-0.89	-4.44	*
G. candidum	GECA06s02276g	similar to Saccharomyces cerevisiae YKL220C FRE2 Ferric reductase and cupric reductase	0.00	-0.45	-0.78	-3.85	*
G. candidum	GECA01s05180g	similar to Saccharomyces cerevisiae YKL220C FRE2 Ferric reductase and cupric reductase	0.00	1.33	-0.19	-0.27	*
G. candidum	GECA01s05213g	similar to Saccharomyces cerevisiae YKL220C FRE2 Ferric reductase and cupric reductase	0.00	1.31	-0.12	0.03	*
G. candidum	GECA19s01132g	similar to Saccharomyces cerevisiae YOR381W FRE3 Ferric reductase, reduces siderophore-bound iron prior to uptake by transporters	0.00	-2.06	-0.46	1.84	*
G. candidum	GECA32s00846g	similar to Saccharomyces cerevisiae YOR384W FRE5 Putative ferric reductase with similarity to Fre2p	0.00	-1.10	-4.88	-2.74	*
G. candidum	GECA16s00164g	similar to Saccharomyces cerevisiae YOR384W FRE5 Putative ferric reductase with similarity to Fre2p	0.00	-0.81	-1.29	0.66	*
G. candidum	GECA13s00879g	similar to Saccharomyces cerevisiae YOL152W FRE7 Putative ferric reductase with similarity to Fre2p	0.00	1.12	1.05	-0.09	*
G. candidum	GECA20s01022g	similar to Saccharomyces cerevisiae YLR047C FRE8 Protein with sequence similarity to iron/copper reductases	0.00	0.00	0.05	-1.47	*
G. candidum	GECA08s00802g	similar to Saccharomyces cerevisiae YLR047C FRE8 Protein with sequence similarity to iron/copper reductases	0.00	0.60	0.43	1.01	*
G. candidum	GECA12s00384g	similar to Saccharomyces cerevisiae YBR207W FTH1 Putative high affinity iron transporter involved in transport of intravacuolar stores of iron	0.00	0.40	0.44	-1.86	*
G. candidum	GECA09s00582g	similar to Saccharomyces cerevisiae YBR207W FTH1 Putative high affinity iron transporter involved in transport of intravacuolar stores of iron	0.00	1.33	3.24	4.39	*
G. candidum	GECA05s03794g	similar to Saccharomyces cerevisiae YER145C FTR1 High affinity iron permease involved in the transport of iron across the plasma membrane	0.00	0.50	-0.72	-1.69	*
G. candidum	GECA26s00230g	similar to Saccharomyces cerevisiae YER145C FTR1 High affinity iron permease involved in the transport of iron across the plasma membrane	0.00	1.23	1.81	0.46	*
G. candidum	GECA01s09173g	similar to Saccharomyces cerevisiae YJL133W MRS3 Iron transporter that mediates Fe2+ transport across the inner mitochondrial membrane	0.00	0.70	0.45	-0.77	
G. candidum	GECA04s00681g	similar to Saccharomyces cerevisiae YKR052C MRS4 Iron transporter of the mitochondrial carrier family	0.00	0.90	1.13	0.33	*
G. candidum	GECA12s01770g	similar to Saccharomyces cerevisiae YEL065W SIT1 Ferrioxamine B transporter	0.00	0.92	0.70	-2.24	*
G. candidum	GECA07s02375g	similar to Saccharomyces cerevisiae YEL065W SIT1 Ferrioxamine B transporter	0.00	1.76	1.51	2.58	*
G. candidum	GECA02s06786g	similar to Saccharomyces cerevisiae YEL065W SIT1 Ferrioxamine B transporter	0.00	1.32	1.79	-1.98	*
G. candidum	GECA10s00736g	similar to Saccharomyces cerevisiae YHR050W SMF2 Divalent metal ion transporter involved in manganese homeostasis	0.00	-1.33	-3.30	-2.04	*
G. candidum	GECA08s02430g	similar to Saccharomyces cerevisiae YHR050W SMF2 Divalent metal ion transporter involved in manganese homeostasis	0.00	-0.09	-0.61	-1.57	*
G. candidum	GECA07s03695g	similar to Saccharomyces cerevisiae YHR050W SMF2 Divalent metal ion transporter involved in manganese homeostasis	0.00	0.05	-0.02	-1.24	*
G. candidum	GECA07s02353g	conserved hypothetical protein. Putative siderophore esterase IroE-like protein	0.00	0.59	0.57	2.83	*
		Fold change calculated from cumulated reads of G. candidum iron transporters:	***0.00***	***0.43***	***0.19***	***-1.42***	
D. hansenii	DEHA2G07986g	similar to uniprot|P38995 Saccharomyces cerevisiae YDR270W CCC2 copper-transporting P-type ATPase	0.00	1.93	1.85	1.89	*
D. hansenii	DEHA2G05082g	similar to Saccharomyces cerevisiae YMR058W FET3 Ferro-O2-oxidoreductase required for high-affinity iron uptake and involved in mediating resistance to copper ion toxicity	0.00	2.21	2.19	1.58	*
D. hansenii	DEHA2C06314g	similar to uniprot|P53746 Saccharomyces cerevisiae YNR060W FRE4 Ferric reductase or uniprot|P36033 Saccharomyces cerevisiae YKL220C FRE2 Ferric reductase	0.00	2.59	2.07	1.58	*
D. hansenii	DEHA2C06248g	weakly similar to uniprot|P53746 Saccharomyces cerevisiae YNR060W FRE4 Ferric reductase	0.00	2.24	2.15	0.97	*
D. hansenii	DEHA2C06292g	weakly similar to uniprot|P53746 Saccharomyces cerevisiae YNR060W FRE4 Ferric reductase	0.00	2.07	1.59	0.67	*
D. hansenii	DEHA2B02772g	weakly similar to uniprot|P53746 Saccharomyces cerevisiae YNR060W FRE4 Ferric reductase	0.00	1.55	1.37	0.17	
D. hansenii	DEHA2B15994g	weakly similar to uniprot|Q08908 Saccharomyces cerevisiae YOR384W FRE5 Putative ferric reductase	0.00	2.80	2.89	3.56	*
D. hansenii	DEHA2D01452g	similar to uniprot|Q12333 Saccharomyces cerevisiae YOL152W FRE7 Putative ferric reductase	0.00	1.34	1.60	1.08	*
D. hansenii	DEHA2C06336g	similar to Saccharomyces cerevisiae YBR207W FTH1 Putative high affinity iron transporter involved in transport of intravacuolar stores of iron	0.00	3.70	3.97	3.67	*
D. hansenii	DEHA2D04554g	similar to Saccharomyces cerevisiae YER145C FTR1 High affinity iron permease involved in the transport of iron across the plasma membrane	0.00	2.49	2.35	2.61	*
D. hansenii	DEHA2E02596g	weakly similar to Saccharomyces cerevisiae YEL065W SIT1 Ferrioxamine B transporter	0.00	3.65	3.55	3.51	*
D. hansenii	DEHA2A14696g	weakly similar to Saccharomyces cerevisiae YEL065W SIT1 Ferrioxamine B transporter	0.00	2.88	2.97	3.12	*
D. hansenii	DEHA2A03806g	weakly similar to Saccharomyces cerevisiae YEL065W SIT1 Ferrioxamine B transporter	0.00	3.59	3.51	2.69	*
D. hansenii	DEHA2B16478g	weakly similar to Saccharomyces cerevisiae YEL065W SIT1 Ferrioxamine B transporter	0.00	3.28	3.35	2.33	*
D. hansenii	DEHA2C05390g	similar to Saccharomyces cerevisiae YEL065W SIT1 Ferrioxamine B transporter	0.00	1.70	1.63	0.48	*
		Fold change calculated from cumulated reads of D. hansenii iron transporters:	***0.00***	***2.21***	***2.17***	***1.72***	
**de novo NAD biosynthesis:**							
G. candidum	GECA24s01088g	similar to Saccharomyces cerevisiae YJR078W BNA2 Putative tryptophan 2,3-dioxygenase or indoleamine 2,3-dioxygenase	0.00	0.56	1.11	3.71	*
G. candidum	GECA06s03288g	similar to Saccharomyces cerevisiae YDR428C BNA7 Formylkynurenine formamidase, involved in the de novo biosynthesis of NAD from tryptophan via kynurenine	0.00	0.22	0.53	4.28	*
G. candidum	GECA02s06456g	similar to Saccharomyces cerevisiae YDR428C BNA7 Formylkynurenine formamidase, involved in the de novo biosynthesis of NAD from tryptophan via kynurenine	0.00	0.17	-0.06	0.55	*
G. candidum	GECA18s02518g	similar to Saccharomyces cerevisiae YBL098W BNA4 Kynurenine 3-mono oxygenase, required for the de novo biosynthesis of NAD from tryptophan via kynurenine	0.00	0.08	-0.10	-1.16	*

G. candidum	GECA06s01847g	similar to Saccharomyces cerevisiae YLR231C BNA5 Kynureninase, required for the de novo biosynthesis of NAD from tryptophan via kynurenine	0.00	-0.14	-0.25	-1.24	*
G. candidum	GECA05s05169g	similar to Saccharomyces cerevisiae YJR025C BNA1 3-hydroxyanthranilic acid dioxygenase, required for the de novo biosynthesis of NAD from tryptophan via kynurenine	0.00	0.34	0.29	0.29	*
G. candidum	GECA05s07149g	similar to Saccharomyces cerevisiae YFR047C BNA6 Quinolinate phosphoribosyl transferase, required for the de novo biosynthesis of NAD from tryptophan via kynurenine	0.00	0.25	-0.22	-2.54	*
G. candidum	GECA12s01638g	similar to Saccharomyces cerevisiae YLR328W NMA1 Nicotinic acid mononucleotide adenylyltransferase	0.00	0.30	0.34	-0.15	*
G. candidum	GECA15s01462g	similar to Saccharomyces cerevisiae YHR074W QNS1 Glutamine-dependent NAD(+) synthetase	0.00	0.27	-0.03	-2.23	*
		Fold change calculated from cumulated reads of G. candidum de novo NAD biosynthesis proteins:	***0.00***	***0.36***	***0.59***	***2.68***	
D. hansenii	DEHA2G12188g	similar to Saccharomyces cerevisiae YJR078W BNA2 Putative tryptophan 2,3-dioxygenase or indoleamine 2,3-dioxygenase	0.00	-0.89	-1.32	-0.26	
D. hansenii	DEHA2C05984g	similar to Saccharomyces cerevisiae YBL098W BNA4 Kynurenine 3-mono oxygenase, required for the de novo biosynthesis of NAD from tryptophan via kynurenine	0.00	0.22	0.34	0.30	
D. hansenii	DEHA2G14080g	similar to Saccharomyces cerevisiae YLR231C BNA5 Kynureninase, required for the de novo biosynthesis of NAD from tryptophan via kynurenine	0.00	0.30	0.48	-0.04	
D. hansenii	DEHA2G23034g	similar to Saccharomyces cerevisiae YJR025C BNA1 3-hydroxyanthranilic acid dioxygenase, required for the de novo biosynthesis of NAD from tryptophan via kynurenine	0.00	0.25	0.43	1.01	*
D. hansenii	DEHA2F26378g	similar to Saccharomyces cerevisiae YFR047C BNA6 Quinolinate phosphoribosyl transferase, required for the de novo biosynthesis of NAD from tryptophan via kynurenine	0.00	0.45	0.29	0.06	
D. hansenii	DEHA2C11704g	similar to Saccharomyces cerevisiae YLR328W NMA1 Nicotinic acid mononucleotide adenylyltransferase	0.00	-0.07	-0.03	0.59	*
D. hansenii	DEHA2A01540g	similar to Saccharomyces cerevisiae YHR074W QNS1 Glutamine-dependent NAD(+) synthetase	0.00	-0.01	0.05	0.06	
		Fold change calculated from cumulated reads of D. hansenii de novo NAD biosynthesis proteins:	***0.00***	***-0.48***	***-0.64***	***0.01***	
**Carbonic anhydrase:**							
G. candidum	GECA05s01473g	similar to Saccharomyces cerevisiae YNL036W NCE103 Carbonic anhydrase	0.00	-0.70	-2.03	-5.04	*
G. candidum	GECA18s00703g	similar to Saccharomyces cerevisiae YNL036W NCE103 Carbonic anhydrase	0.00	0.17	-0.02	-0.69	
D. hansenii	DEHA2A12430g	similar to uniprot|P53615 Saccharomyces cerevisiae YNL036W NCE103 Carbonic anhydrase	0.00	-0.76	-0.52	-3.20	*
**Plasma membrane H+-ATPase:**							
G. candidum	GECA07s01099g	similar to Saccharomyces cerevisiae YGL008C PMA1 Plasma membrane H+-ATPase	0.00	-0.29	-1.04	-2.58	*
D. hansenii	DEHA2A08800g	highly similar to uniprot|P05030 Saccharomyces cerevisiae YGL008C PMA1 Plasma membrane H+-ATPase	0.00	-0.13	-0.21	-1.20	*
