# Supplementary material for: Investigation of the Activity of the Microorganisms in a Reblochon-Style Cheese by Metatranscriptomic Analysis
Source: Front Microbiol. 2016 Apr 20;7:536. doi: 10.3389/fmicb.2016.00536 (PMC4837152; doi:10.3389/fmicb.2016.00536)
Supplement: Supplementary file 3 [file Table3.PDF]

Supplementary Table 3. Biochemical changes during cheese ripening. Measurements were performed in a previous study (Castelotte et al., 2015), except lipolysis, which was measured as described by Mouillet et al. (1981).

	Day 5	Day 14	Day 19	Day 35
Lactate (g/100 g)	0.78	0.55	0.45	0.13
Galactose (g/100 g)	0.48	0.11	0.06	0.00
Lactose (g/100 g)	0.02	0.00	0.00	0.00
Ammonia (mg/100 g)	0.00	0.00	27.7	148. 7
Non-protein nitrogen (gN/100 g)	0.28	0.34	0.41	2.00
Lipolysis degree (mg KOH/g)	4.94	6.06	7.95	15.40
Rind pH	5.70	5.80	5.94	6.77

Mouillet, L., F.M. Luquet, H. Nicod, J.F. Boudier, and H. Mahieu. 1981. La lipolyse des laits. Etude d’une méthode rapide de mesure. Lait. 61: 171–186
