# Supplementary material for: Investigation of the Activity of the Microorganisms in a Reblochon-Style Cheese by Metatranscriptomic Analysis
Source: Front Microbiol. 2016 Apr 20;7:536. doi: 10.3389/fmicb.2016.00536 (PMC4837152; doi:10.3389/fmicb.2016.00536)
Supplement: Supplementary file 6 [file Image2.PDF]

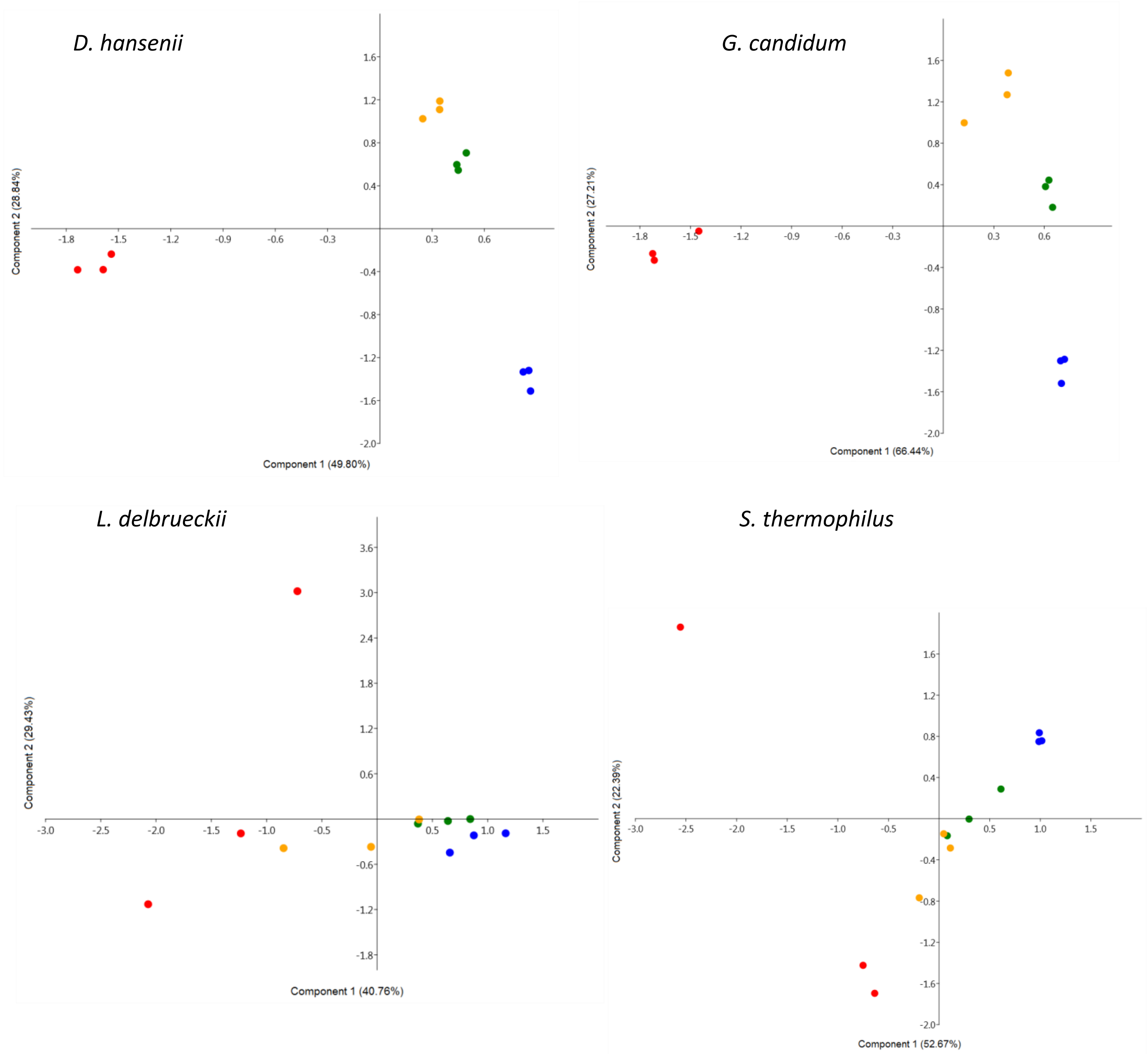

Supplementary Figure 2. Principal component analysis of the RNA-seq data normalized against the corresponding species. The three cheeses replicates are represented in blue (day 5), green (day 14), orange (day 19) and red (day 35).
